# Surface Hopping Nested Instances Training Set for Excited-state Learning

**DOI:** 10.1038/s41597-025-05443-5

**Published:** 2025-07-26

**Authors:** Robin Curth, Theodor E. Röhrkasten, Carolin Müller, Julia Westermayr

**Affiliations:** 1 Leipzig University, Wilhelm Ostwald Institute for Physical and Theoretical Chemistry, Linnéstraße 2, Leipzig, 04103 Germany; 2 Center for Scalable Data Analytics and Artificial Intelligence (ScaDS.AI), Dresden/Leipzig, Humboldtstraße 25, Leipzig, 04105 Germany; 3 Friedrich-Alexander-Universität Erlangen-Nürnberg, Computer-Chemistry-Center, Nägelsbachstraße 25, Erlangen, 91052 Germany

**Keywords:** Quantum chemistry, Photochemistry, Computational chemistry

## Abstract

Theoretical studies of molecular photochemistry and photophysics are essential for understanding fundamental natural processes but rely on computationally demanding quantum chemical calculations. This complexity limits both direct simulations and the development of machine learning (ML) models trained on this data. To address this, we introduce SHNITSEL, a data repository containing 418,870 ***ab-initio*** data points of nine organic molecules in their ground and electronically excited states. Each data point includes high-accuracy quantum chemical properties such as energies, forces, and dipole moments in the ground state and electronically excited singlet or triplet states as well as properties that arise from the coupling of electronic states, namely nonadiabatic couplings, transition dipoles, or spin-orbit couplings. Generated with state-of-the-art methods, SHNITSEL provides a robust benchmark for ML models and facilitates the development of ML-based approaches for excited state properties.

## Background & Summary

Light-induced processes are crucial in natural phenomena^1–5^ and the development of sustainable chemical transformations^6–8^. In the latter context understanding the underlying electronic and nuclear dynamics is essential. However, the ultrafast nature of photo-induced phenomena complicates their direct experimental characterization, often resulting in speculative structure-property relationships when relying solely on experimental data. To address these challenges, quantum chemistry is an indispensable tool. In combination with nonadiabatic molecular dynamics (NAMD) simulations, it enables the study of time-dependent excited-state evolution with atomistic resolution. However, high computational costs limit their applicability to small systems (≤100 atoms) and femto- to picoseconds timescales^9–11^.

Machine learning (ML) models trained on quantum-chemical data offer a promising solution, enabling efficient predictions of electronic properties at much lower computational costs^10,12–14^. By decoupling electronic structure calculations from dynamics simulations, ML-based PES representations enable the study of larger systems and longer timescales, significantly advancing excited-state simulations^10,15,16^.

Although ML algorithms for fitting ground-state potential energy surfaces (PESs) and properties are already very advanced (see references^17–19^ for recent reviews on this topic) with foundational models such as MACE-OFF^20^ or MACE-MP0^21^, the development of equivalent models for excited states remains significantly less advanced^14,22,23^. Current excited state ML approaches are restricted to individual molecules^9,10,13,16,24^ or are tailored to specific molecular classes^14^. First studies toward transferability in excited-state PESs are based on the MACE foundational model parameters, fine-tuned to excited states in vacuum^22^ and under the influence of external fields^23^. Key challenges in modeling excited states include the need to fit multiple electronic states with different spin multiplicities and to handle non-smooth properties, such nonadiabatic couplings^10,11,13,24^. Furthermore, the development of ML models for excited-state properties is hindered by the limited availability of high-quality, standardized datasets. Although extensive data repositories exist for ground-state PESs^25–29^, excited-state datasets are primarily restricted to the adiabatic approximation and single-reference methods, particularly time-dependent density functional theory (TDDFT)^30–34^. Though TDDFT is computationally efficient, it struggles to describe nonadiabatic processes^35^, which require multi-reference methods^36^. These methods are computationally demanding and rely on expert knowledge, such as the selection of active spaces, making large-scale dataset generation difficult. Consequently, the lack of diverse and high-fidelity training data remains a major challenge in developing generalizable ML models for excited states^9,36^.

**Fig. 1:**
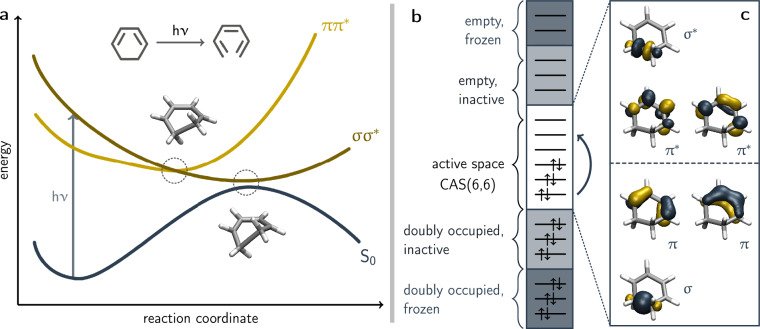
Schematic representation of the potential energy curves for the bond dissociation reaction of cyclohexa-1,3-diene to hexa-1,3,5-triene upon light activation (**a**). The geometries at the conical intersections between the σσ^*^ (${{\mathsf{S}}}_{{\mathsf{2}}}$) and ππ^*^ (${{\mathsf{S}}}_{{\mathsf{1}}}$) states, as well as between the ππ^*^ and the ground state (${{\mathsf{S}}}_{{\mathsf{0}}}$), are highlighted (3D-structures are taken from reference^40^). These intersections are crucial for understanding the nonadiabatic dynamics of the system. Schematic representation of the complete active space self-consistent field (CASSCF) method (**b**). In this method, orbitals are divided into three categories: frozen, inactive, and active. Frozen orbitals remain unchanged during the computation. Inactive orbitals are optimized with respect to the coefficients of the atomic orbitals forming the molecular orbitals. Active orbitals are treated with the highest level of theory, where a full configuration interaction (Full-CI) calculation is performed within the active space. An example active space, consisting of six active electrons in six orbitals (175 configuration state functions), is shown for **R02** (**c**). For **R02**, the active space includes two occupied *π* orbitals, their corresponding 2π^*^ orbitals, and a pair of *σ* and σ^*^ orbitals, which describe the bond dissociation of cyclohexa-1,3-diene upon light absorption. Active orbitals were calculated at the SA(3)-CAS(6,6)SCF/cc-pVDZ level of theory using *OpenMolcas*.

To advance the development and validation of ML models for excited state dynamics, we introduce the **S**urface **H**opping **N**ested **I**nstances **T**raining **S**et for **E**xcited-State **L**earning (SHNITSEL), a comprehensive data repository designed to develop and benchmark excited state methods. It contains data sets for nine organic molecules (see Table 1) that were selected to represent a broad range of photochemical behaviors. SHNITSEL includes a series of alkenes^37,38^ – ethene (**A01**), propene (**A02**), and 2-butene (**A03**) – whose photoinduced processes are monitored in the first excited singlet state (${{\mathsf{S}}}_{{\mathsf{1}}}$). These molecules exhibit characteristic surface switches at the 90° torsion angle around the alkene bond, whereas **A03** can undergo *E/Z*-isomerization. The data set also includes organic molecules with ring structures, namely fulvene (**R01**), 1,3-cyclohexadiene (**R02**), and tyrosine (**R03**). **R01** has an extremely short excited state lifetime due to a readily accessible planar conical intersection along the methylene torsion coordinate^39^. **R02** undergoes a light-induced ring-opening upon population of a σσ^*^ state (*cf*. Fig. 1)^40^, while **R03**^41^ is known as the first biological system where hydrogen atom roaming was observed^15^. Among these, **A01** and **R01** are commonly used in molecular Tully models for benchmarking surface hopping and NAMD simulations^42^. In addition, the data set covers molecules with distinct excited-state behaviors. The methylenimmonium cation (**I01**) exhibits ultrafast internal conversion following excitation^43,44^, whereas methanethione (**T01**) undergoes slow singlet-to-triplet population transfer^12,45^. Diiodomethane (**H01**) is included to represent systems with strong spin-orbit coupling effects and iodine dissociation dynamics^46^.

**Table 1:** Summary of the IUPAC names, SMILES, InChI and 3D structures of the molecules represented in the SHNITSEL data repository and their respective SHNITSEL dataset identifiers (first column).

Index	Class	IUPAC name	Molecular formula	SMILES	InChI	3D structure
**A01**	alkene	ethene	C_2_H_4_	C=C	InChI=1S/C2H4/c1-2/h1-2H2	
**A02**	alkene	propene	C_3_H_6_	CC=C	InChI=1S/C3H6/c1-3-2/h3H,1H2,2H3	
**A03**	alkene	2-butene	C_4_H_8_	CC=CC	InChI=1S/C4H8/c1-3-4-2/h3-4H,1-2H3	
**R01**	ring (aromatic)	fulvene	C_6_H_6_	C=C1C=CC=C1	InChI=1S/C6H6/c1-6-4-2-3-5-6/h2-5H,1H2	
**R02**	ring (alkene)	1,3-cyclohexadiene	C_6_H_8_	C1CC=CC=C1	InChI=1S/C6H8/c1-2-4-6-5-3-1/h1-4H,5-6H2	
**R03**	ring (amino acid)	2-amino-3-(4-hydroxyphenyl)propanoic acid	C_9_H_11_NO_3_	C1=CC(=CC=C1CC(C(=O)O)N)O	InChI=1S/C9H11NO3/c10-8(9(12)13)5-6-1-3-7(11)4-2-6/h1-4,8,11H,5,10H2,(H,12,13)	
**I01**	imine	methylideneazanium	CH_4_N^+^	C=[NH2+]	InChI=1S/CH3N/c1-2/h2H,1H2/p+1	
**H01**	halide	diiodimethane	CH_2_I_2_	C(I)I	InChI=1S/CH2I2/c2-1-3/h1H2	
**T01**	thione	methanethione	CH_2_S	C=S	InChI=1S/CH2S/c1-2/h1H2

These respective photochemistry and -physics are represented in terms of electronic properties of the singlet ground state as well as electronically excited singlet and triplet states described by the energies (*E*_*i*_), forces (**F**_*i*_), and permanent dipole moments (*μ*_*i*_) in the respective states (*i*); and the properties that arise form the coupling of them, namely transition dipole moments (*μ*_*ij*_), nonadiabatic couplings (**NAC**_*ij*_) and spin-orbit couplings (**SOC**_*ij*_). These properties are computed at multi-reference ab initio level of theory for the SHNITSEL molecules, with a main fraction (70%) on complete-active-space self-consitent field level of theory.

To the best of our knowledge, the SHNITSEL dataset is the first to provide benchmark datasets for fitting excited states of molecules across different spin multiplicities, covering not only energies but also forces and other key properties required for excited-state molecular dynamics simulations. We believe this facilitates the creation of generalizable ML models^47,48^, advancing excited-state dynamics research.

## Methods

Accurately modeling excited-state PESs presents significant challenges due to the complex electronic structure landscape. Unlike the ground state, which is often well-separated from higher electronic states, excited states frequently cluster within narrow energy ranges, exhibiting strong state mixing and reordering upon small nuclear displacements (*cf*. Fig. 1a). The core task in excited-state simulations is the electronic structure calculation, which determines potential energy and properties such as transition dipole moments for fixed nuclear geometries. These calculations rely on either single-reference methods (like Hartree Fock or TDDFT) or multireference approaches that explicitly account for strong electron correlation effects. The SHNITSEL dataset is primarily constructed using *post*-Hartree-Fock methods from the latter category, which offer a balanced treatment of static and dynamic electron correlation, particularly in systems with pronounced multireference character. These methods are essential for accurately capturing electronic state mixing and near-degeneracies, where single-reference approaches often break down. In the following section, we provide a concise overview of the key methods with which the SHNITSEL data was generated, and subsequently, we outline our data generation workflow focusing on the generation of data using quantum chemistry and ML.

### Quantum Chemistry Reference Methods

#### Variational *post*-Hartree-Fock methods

These methods are based on the variational principle to find the best approximation of the wavefunction that minimizes the energy. Among them, complete-active-space self-consistent-field (CASSCF) is a widely used multireference method, which was utilized in the generation of the data of all nine molecules reported in SHNITSEL. CASSCF is built upon full configuration interaction (Full-CI), where the total *N*-electron wavefunction for state ${\mathsf{v}}$ ($\left|{\Psi }_{{\mathsf{v}}}\right\rangle$) is expanded in terms of configuration state functions, here expressed as Slater determinants ($\left|{\phi }_{{\mathsf{k}}}\right\rangle$):

1 $$\left|{\Psi }_{{\mathsf{v}}}\right\rangle =\sum _{{\mathsf{k}}}{{\mathsf{C}}}_{{\mathsf{kv}}}\left|{\phi }_{{\mathsf{k}}}\right\rangle =\sum _{{\mathsf{k}}}\sum _{{\mathsf{l}}}{{\mathsf{C}}}_{{\mathsf{kv}}}{{\mathsf{b}}}_{{\mathsf{il}}}{\varphi }_{{\mathsf{i}}}.$$

Each determinant is constructed from orthonormal molecular orbitals (${\varphi }_{{\mathsf{i}}}$), and the expansion coefficients ${{\mathsf{C}}}_{{\mathsf{kv}}}$ describe their contribution. Full-CI considers all possible configurations, but its computational cost scales exponentially with system size, making it impractical for real molecules. To overcome this limitation, CASSCF approximates Full-CI by defining an active space, denoted CASSCF(n,m), where n is the number of active electrons and m the number of active orbitals. Full-CI is performed only within this active space, while inactive orbitals (core and virtual) remain doubly occupied or unoccupied (see CAS(6,6) example for **R02** in Fig. 1). Unlike configuration interaction (CI) methods, which use orbitals from a Hartree-Fock reference, CASSCF optimizes both the molecular orbitals (${{\mathsf{b}}}_{{\mathsf{il}}}$) and the configuration expansion coefficients simultaneously, ensuring a self-consistent description of electron correlation.

Notably, 73% of the whole SHNITSEL data was obtained at the CASSCF level of theory. Beyond CASSCF, data obtained at multireference CI singles and doubles (MR-CISD^49^) level of theory is reported in SHNITSEL. In detail, for **A01** and **I01** the CASSCF(6,4) wavefunction was used as a reference and MR-CISD was used to introduce additional single and double excitations outside the active space to further improve the correlation treatment.

#### Perturbative *post*-Hartree-Fock methods

Perturbative methods enhance reference wavefunctions, such as CASSCF, by introducing correlation corrections. In the SHNITSEL dataset, two such approaches were employed. Complete Active Space Perturbation Theory (CASPT2)^50,51^ was used for datasets **R02**^40^, **R03**^15^, and **H01**^46^. By incorporating dynamic electron correlation through second-order perturbation theory, CASPT2 can significantly improve the accuracy of the excited-state PESs. Additionally, Algebraic Diagrammatic Construction (ADC(2))^52^ was applied in the data generation of **R03**^15^. Based on second-order Møller-Plesset perturbation theory (MP2)^53,54^, ADC(2) employs a Green’s function formalism to describe excited-state properties through polarization propagation. For further methodological details, we refer to the original references^49,52–54^.

### SHNITSEL data

The SHNITSEL dataset comprises a total of 418,870 molecular geometries along with their corresponding electronic properties. The included data were generated using a variety of reference electronic structure methods and sampling strategies, reflecting the heterogeneity of their original sources and targeted applications. A substantial majority (97%) of the data was computed at the multi-reference level of theory. In contrast, a smaller fraction (3%), corresponding exclusively to dataset **R03**, was obtained using a single-reference method.

Generally, in the SHNITSEL dataset^55^ the data is categorized into **dynamic** and **static data** based on its origin and purpose. A summary of the data distribution across different molecules and between static and dynamic datasets is provided in Table 3 and visualized in Fig. 3. **Static data** consists of sampled molecular structures without time-dependent information, covering relevant vibrational and conformational spaces. Sampling strategies include:

- 2D grid sampling (**A01**–**A03**^13,37^): Structures generated by rigid 2D scan of a torsion angle and bond distance (each 3,731 data points).
- Wigner sampling (**R01**^56^, **R02**^40^, **R03**^15,41,57^, **I01**^38^, and **T01**^12^): Initial conditions drawn from a quantum harmonic oscillator approximation.
- Adaptive sampling^43,58^ (**A01**^38^, **R01,** **R03**^15,57^, **I01**^38^): structures added from surface hopping trajectories *via* active learning (*cf*. the Data Sources and Data Generation sections for more details).

**Dynamic data** originates from surface hopping NAMD simulations and captures the evolution of molecular structures and properties over time as they propagate on PESs according to Newton’s equations of motion. The respective datasets, covering **A01**–**A03**^13^, **R02**^40^, and **I01**^38^, were generated *via* surface hopping simulations, with methodological details provided in the respective publications for **R02**^40^ and **I01**^38^ (see Table 3), and described herein for **A01–A03** (*cf*. Data Generation section).

### Data Sources

The SHNITSEL dataset is primarily composed of data compiled from previously published sources^12,13,15,37,38,40,41,43,46^. An overview of the corresponding publications and original data sources is provided in Table 2. However, not all data was published with the corresponding resource and has been provided specifically for the purpose of this database. Data for **H01** and **T01** were not published with the original publications^12,46^, but were provided by the authors for this database. The data have been incorporated without altering their content, aside from standardizing the file format. In the following, we briefly outline the adaptive sampling approach, which was employed to diversify the datasets of **A01 (a),** **I01** and **R03** in the original publications^15,38^.

**Table 2:** Overview of the data sources and original publications of previously published or described data included in SHNITSEL.

molecule-ID	data source	data description
static data		
**A01 (a)**	^38^: Supporting Information	^38^
**A01–A03** (grid)	^37^: Zenodo repository	^13^
**A01 (b), A02, A03**	^37^: Zenodo repository	^13^
**R03**	^41^: Figshare repository	^15^
**I01**	^43^: Supporting Information	^43^
**H01**	data provided by Weinacht *et al*.	^46^
**T01**	data provided by Marquetand *et al*.	^12^
trajectory data		
**A03**	data provided by Müller *et al*.	^13^
**R02**	data provided by Polyak *et al*.	^40^
**I01**	data provided by Marquetand *et al*.	^43^

The static data of **A01 (a)** was derived from the data of **I01**^38^ by replacing the nitrogen atom with carbon while maintaining the same level of theory (MR-CISD/CASSCF(6,4)/aug-cc-pVDZ), enabling the exploration of transferable ML models for excited states. For **I01**, SchNarc models trained on Wigner-sampled data were used to identify under-sampled PES regions during NAMD simulations^38^. Quantum chemistry calculations were performed for high-uncertainty points, iteratively refining the dataset – as described in the original work by Westermayr and co-authors^38^.

In case of **R03**, single-reference data at ADC(2) level of theory was supplemented with *ad-hoc* corrections in regions characterized by large interatomic distances, where single-reference approaches are known to be unreliable and provide wrong physics^15^. To this end, the maximum X-H bond distances that can be reasonably described using the single-reference method ADC(2) were determined by comparing scans along various reaction coordinates using ADC(2), MP2, and multi-reference methods like CASPT2(12,11). The specific thresholds and further details can be found in the original publication^15^. The physically inaccurate ADC(2) has been refined based on multi-reference methods to describe the system correctly. The SHNITSEL dataset incorporates this complete dataset from reference^15^.

Data for **T01** were presented in reference^12^, but not published with the original publication and thus provided for this database. They were based on Wigner sampling and surface hopping molecular dynamics using SHARC and a linear vibronic coupling model that was based on CASSCF(6,5)/def2-SVP with parameters for the linear vibronic coupling model and details about surface hopping dynamics specified in the original publication.

### Data Generation

The datasets of **R01,** **A01,** **A02** and **A03** are unpublished and newly introduced herein and are based on surface hopping molecular dynamics simulations that were conducted following the standard procedure in SHARC and excitation to the first excited singlet state (${{\mathsf{S}}}_{{\mathsf{1}}}$). The data generation pipelines for each dataset are detailed in the subsequent sections.

#### R01 (static)

**R01** belongs to the collection of molecular Tully models and has served the evaluation of a new coupling approximation for surface hopping simulations^56,59^. The photodynamics of **R01** is characterized by a stepwise population decrease of the first excited state. This occurrence is due to reflection. The ground state and the first excited singlet state are almost degenerate in the beginning of the dynamics. This degeneracy is lifted shortly after an avoided crossing. When the population is transferred at the intersection seam, it can be reflected back.

The unpublished **R01** dataset, newly introduced here, was generated from 18 surface-hopping simulations collected over 75 fs at SA(2)-CASSCF(6,6)/6-31G* level of theory as introduced in reference^56^. The initial conditions were obtained from reference^56^ and we followed the same procedure for dynamics simulations as described in this study^56^, which means that the velocities of the initial conditions were set to 0. ML models, *i.e*., SchNarc models^12^ were trained on *E*_*i*_ and **F**_*i*_ of the ground- (${{\mathsf{S}}}_{{\mathsf{0}}}$) and first excited state (${{\mathsf{S}}}_{{\mathsf{1}}}$) and the nonadiabatic couplings between these two states (**NAC**_01_), with four adaptive sampling iterations (1,000 trajectories each). In addition, 1,000 data points from a Wigner distribution and 9,960 data points from optimizations of conical intersections were used to adapt the training set. Starting points for conical intersection optimizations were hopping geometries, *i.e*., geometries visited one time step before or after a hop between two states occurred in the dynamics. For each data point that was added to the training set, the difference to geometries in the training set was computed to ensure that no data points were very similar to each other. To ensure diversity, we computed the mean absolute error of distances to evaluate structural similarity geometries and excluded all structures with mean absolute errors below 0.05 Å.

#### A01–A03 (dynamic)

The photoinduced gas-phase dynamics of alkenes **A01–A03** were simulated using SHARC 3.0, employing non-adiabatic molecular dynamics based on Tully’s fewest switches algorithm^60^. Nuclei were treated as classical point particles governed by Newton’s equations of motion, and propagated *via* the Velocity-Verlet algorithm^61^ with a time step of 0.5 fs. Energy gradients were computed analytically at each step. Electronic structure was described using the complete active space self-consistent field (CASSCF) method^62,63^, with state averaging over the three lowest singlet states (${{\mathsf{S}}}_{0,1,2}$). Single-point SA(3)-CASSCF(2,2) calculations were performed at each time step using *OpenMolcas*^64^ with the cc-pVDZ basis set^65^. The active space consisted of two electrons in two orbitals (*π*, π^*^) localized on the central alkene bond. Decoherence effects were accounted for using the energy-based decoherence correction (EDC) by Granucci *et al*.^66^ with *α* = 0.1 Hartree.

Initial conditions for **A01–A03** were derived from static geometries in XAlkeneDB^37^, reused here as static datasets **A01 (b),** **A02**, and **A03**. Details on their preparation are provided in reference^13^. In brief, initial geometries were generated *via* Wigner sampling^67,68^ based on PBE0^69,70^/def2-TZVP^71^/D3BJ^72^-optimized structures. For **A03**, both *E*- and *Z*-isomers were sampled, while **A01** and **A02** were considered as single conformers. Each equilibrium structure yielded 999 displaced geometries, resulting in 1,000 samples per compound. For these initial geometries, the electronic properties of the three lowest singlet states and their couplings were calculated at the SA(3)/CASSCF(2,2)/cc-pVDZ level using *OpenMolcas*. Based on the initial conditions, surface-hopping trajectories were initiated in the ${{\mathsf{S}}}_{{\mathsf{1}}}$ state for each system: 438 for **A01**, 430 for **A02**, and 200 for the *Z*-isomer of **A03**. These trajectories were stochastically selected according to the excitation energies and oscillator strengths of the ${{\mathsf{S}}}_{0}\to {{\mathsf{S}}}_{1}$ transition^73^.

In the SHNITSEL dataset, trajectory points were retained only if the changes in kinetic and potential energy between time steps were below 0.7 eV, and the total energy variation remained under 0.1 eV. After applying these quality filters, the SHNITSEL dataset includes 297, 84, and 87 validated trajectories for **A01,** **A02**, and **A03**, respectively, comprising a total of 62,031, 13,899, and 20,770 dynamic data points. The longest trajectories extend up to 200 fs for **A01** and **A02**, and up to 150 fs for **A03**. The latter includes the data of *Z*-to-*E*-photoisomerization (12%, filtered based on $\varphi ({\mathsf{CC}}={\mathsf{CC}})\ge 15{0}^{\circ }$) and trajectories showing no isomerization.

### Dataset Labels

SHNITSEL is a dataset that stores various quantum chemical properties associated with different data types (such as floats, vectors, matrices, and tensors) for a given molecular conformation. The names of these properties and their corresponding dimensions are summarized in Table 4 and in the following the definition of nonadiabatic couplings (NACs), spin-orbit couplings (SOCs) and (transition) dipole moments (*μ*_*i*_, *μ*_*ij*_) are described.

#### Nonadiabatic couplings (NACs)

NACs couple states of same multiplicity. The NACs between two states, *i* and *j*, obey the following relation: *NAC*_*ij*_ = − *NAC*_*ji*_ and are zero if *i* = *j*.

They are inversely proportional to the energy gap of the respective potentials *i* and *j*:

2 $${{\bf{NAC}}}_{ij}\approx \frac{1}{{E}_{i}-{E}_{j}}\left\langle {\Psi }_{i}\right|\frac{\partial {\widehat{H}}_{el}}{\partial {\bf{R}}}\left|{\Psi }_{j}\right\rangle $$

with **R** referring to the nuclear positions. Ψ_*i*_ and Ψ_*j*_ are the electronic wave functions of states *i* and *j*, respectively, and ${\widehat{H}}_{el}$ is the electronic Hamiltonian. Due to the inverse proportionality of NACs to the energy gap, *E*_*i*_ − *E*_*j*_, of states *i* and *j*, they are singular at avoided crossing points of the respective PESs where *E*_*i*_ = *E*_*j*_. Further, the couplings are close to zero elsewhere. Thus, the couplings can be transformed to “smooth” couplings when training ML models by multiplication with the corresponding energy gap.

#### Spin-orbit couplings (SOCs)

The SOCs between the triplet states and between the singlet and triplet states result in a mixing of the states. There are no SOCs between singlet and singlet states. The couplings between two states $\left|{\Psi }_{i}\right\rangle$ and $\left|{\Psi }_{j}\right\rangle$ is given by the matrix elements^74^: $\left\langle {\Psi }_{i}\right|{H}_{SO}\left|{\Psi }_{j}\right\rangle$. The spin-mixed states can be obtained by diagonalization of the SOC-matrix. The resulting basis is often called the diagonal or spectroscopic basis^75,76^ with zeros as off-diagonal elements. Due to the hermitian nature of the Hamiltonian, the following relation can be used for SOCs: $SO{C}_{ij}=SO{C}_{ji}^{\ast }$.

#### Dipole moments

The permanent dipole moments are related to the atomic partial charges, *q*_*ia*_, of state *i* and atom *a* of a molecule, and are often computed via Hirshfeld or Mulliken charges using the charge model^77^:

3 $${\mu }_{i}=\mathop{\sum }\limits_{a}^{{N}_{A}}{q}_{ia}\cdot {{\bf{r}}}_{a}^{COM}.$$

${{\bf{r}}}_{a}^{COM}$ refers to the distance of an atom *a* to the center of mass (COM) of the molecule.

In contrast, for the excited states, the dipole operator is applied to compute the dipole moments:

4 $${\mu }_{ij}=\langle {\Psi }_{i}| \widehat{\mu }| {\Psi }_{j}\rangle .$$

Thus, the permanent (*i* = *j*) and transition (*i* ≠ *j*) dipole moments in this training set are not related to any partitioning scheme, such as Hirshfeld^78^ or Mulliken^77^, and their accuracy is the same as for any other property reported in the dataset. When modelling dipole moment vectors, the charge scheme of reference^79^ (equation (4)) has been shown very favorable and has allowed to infer latent partial charges, which are not known by a learning algorithm and have been shown accurate when compared to Mulliken or Hirshfeld charges^38,79,80^.

## Data Records

**Fig. 2:**
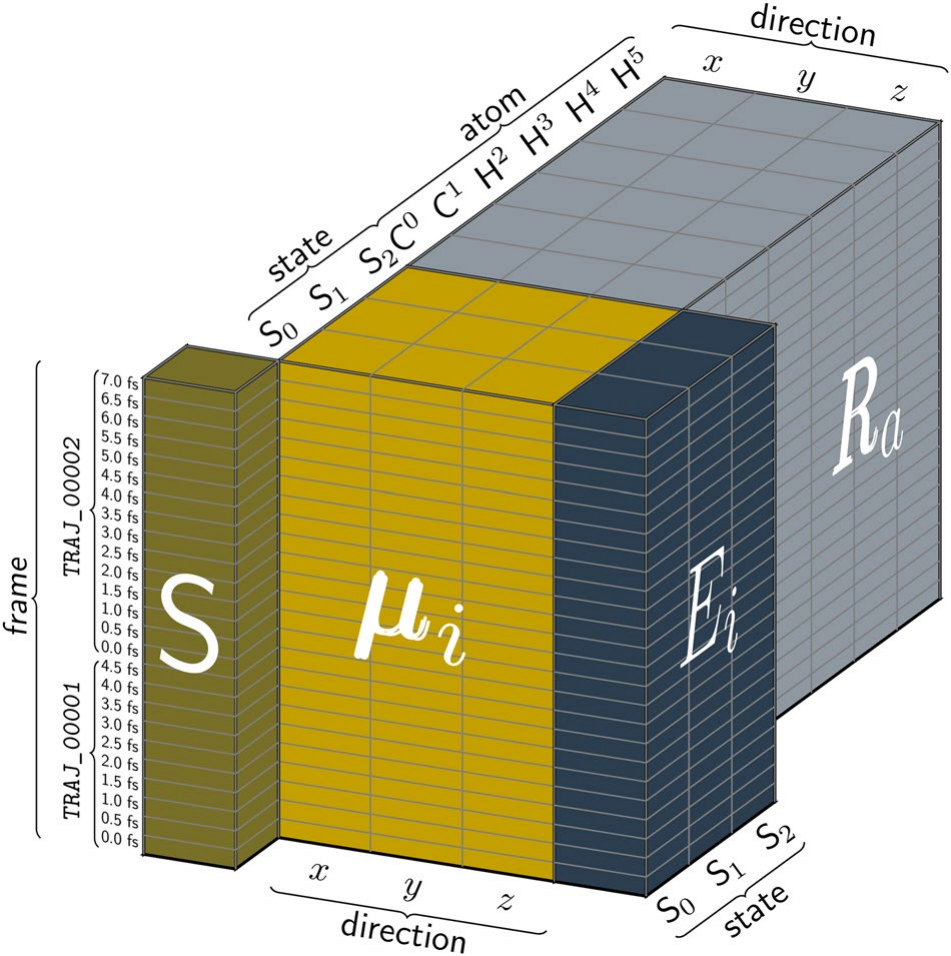
Schematic illustration of the data structure of the SHNITSEL datasets. The figure represents the DataArrays for active state (*S*, 1-dimensional), energies (*E*_*i*_, 2D), permanent dipoles (*μ*_*i*_, 3D) and atom positions (**R**_*a*_) as cuboids, which are combined to make a Dataset. If dimensions are shared between two variables, they can be shown sharing an edge or a face. This is transitive, so all pictured variables share the frame dimension shown on *S*. Energies and dipoles have frame and state dimensions in common, and so share a face. Similarly, dipoles and atom positions share a face due to shared frame and direction dimensions. The frame dimension has two levels (implemented using a MultiIndex): trajectory ID and time.

The SHNITSEL dataset is available on Zenodo^55^ (10.5281/zenodo.15482819). For each molecule represented in SHNITSEL, a single zip archive file bundles the corresponding datasets into one file. Filenames are composed of the specific molecule identifier (*e.g.* **A01**), followed by the total number of datapoints. All datasets are stored in NETCDF format. NetCDF4, encapsulated within HDF5, serves as the preferred storage format, ensuring scalability and compatibility with large datasets. These datasets include molecular structures represented in Cartesian coordinates along with several key quantum chemical properties (see section Dataset Labels). An overview of the computed properties, reference methods, and the number of singlet (*N*_*S*_) and triplet (*N*_*T*_) states considered for each molecule is provided in Table 3. The data structure is illustrated in Fig. 2.

**Table 3:** Summary of datasets in the SHNITSEL data repository.

Molecule	#Data	Reference method	*N*_*S*_/*N*_*T*_	Available properties						Data Source
				*E*_*i*_	F_*i*_	*μ*_*i*_	*μ*_*ij*_	NACs	SOCs	
**A01** (a)	3,969	MR-CISD/aug-cc-pVDZ & SA(3)-CASSCF(6,4)/cc-pVDZ	3/0	*✓*	*✓*	*✓*	*✓*	*✓*	✗	^38^
**A01** (b)	5,999	SA(3)-CASSCF(2,2)/cc-pVDZ	3/0	*✓*	*✓*	*✓*	*✓*	*✓*	✗	^37^
**A01** (grid)	3,731		3/0	*✓*	*✓*	*✓*	*✓*	*✓*	✗	^37^
**A01** (traj)	62,031		3/0	*✓*	*✓*	*✓*	*✓*	*✓*	✗	–
**A02**	6,000	SA(3)-CASSCF(2,2)/cc-pVDZ	3/0	*✓*	*✓*	*✓*	*✓*	*✓*	✗	^37^
**A02** (grid)	3,731		3/0	*✓*	*✓*	*✓*	*✓*	*✓*	✗	^37^
**A02** (traj)	13,899		3/0	*✓*	*✓*	*✓*	*✓*	*✓*	✗	–
**A03**	13,000	SA(3)-CASSCF(2,2)/cc-pVDZ	3/0	*✓*	*✓*	*✓*	*✓*	*✓*	✗	^37^
**A03** (grid)	3,731		3/0	*✓*	*✓*	*✓*	*✓*	*✓*	✗	^37^
**A03** (traj)	36,579		3/0	*✓*	*✓*	*✓*	*✓*	*✓*	✗	^13^^*^
**R01**	20,032	SA(2)-CASSCF(6,6)/6-31G*	2/0	*✓*	*✓*	*✓*	*✓*	*✓*	✗	–
**R02**	92,808	XMS-CASPT2(6,6)/cc-pVDZ	3/0	*✓*	*✓*	*✓*	*✓*	✗	✗	^40^^*^
**R03**	17,265	ADC(2)/cc-pVDZ & ad-hoc data based on ADC(2) and CASPT2(12,11)/ano-rcc-pVDZ	5/8	*✓*	*✓*	*✓*	*✓*	✗	*✓*	^41^
**I01**	4,000	MR-CISD/aug-cc-pVDZ &	3/0	*✓*	*✓*	*✓*	*✓*	*✓*	✗	^43^
**I01** (traj)	39,365	SA(3)-CASSCF(6,4)/cc-pVDZ	3/0	*✓*	*✓*	*✓*	*✓*	*✓*	✗	^43^*
**H01**	60,856	SA(5/4)-CASPT2(12,8)/ano-rcc-VDZP	5/4	*✓*	*✓*	*✓*	*✓*	✗	*✓*	^46^*
**T01**	4,855	SA(2/2)-CASSCF(6,5)/def2SVP	2/2	*✓*	*✓*	*✓*	*✓*	✗	*✓*	^12^*

The data sources column lists openly available datasets and publications, where the data was obtained upon request (marked with an asterisk).

Table S1 shows the name of the entry and its dimension, *i.e*., how many values one entry contains. NACs, dipole moments, forces, and atomic positions are vectorial properties. The last dimension refers to x, y and z directions. Energies are reported in the spin-adiabatic basis^75^, which is the direct output of a quantum chemistry calculation. The triplet states in this basis degenerate, but can be transferred to spin-mixed states using the SOCs.

**Fig. 3:**
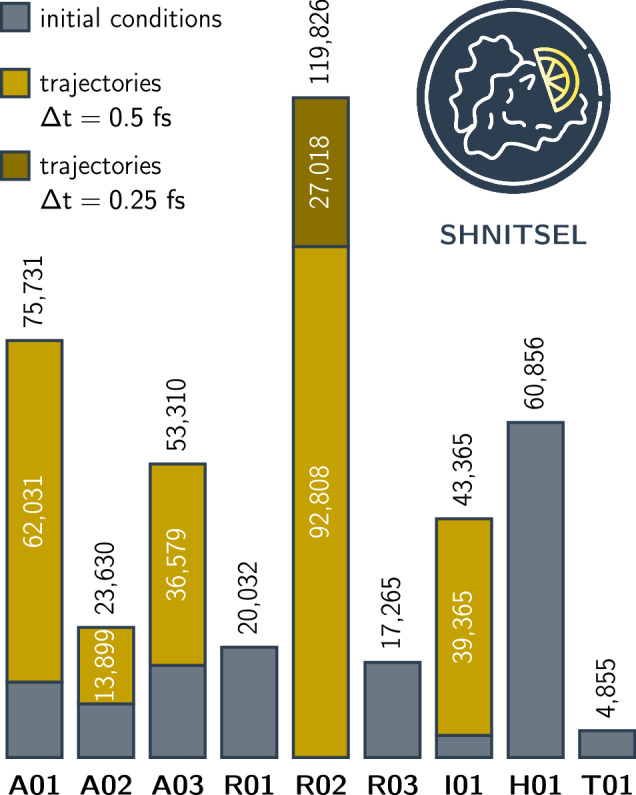
Distribution of the 418,870 data points across the nine molecules in the SHNITSEL dataset, categorized into static (blue bars) and dynamic (yellow shaded bars) data points. Static data points correspond to initial conditions, while dynamic data points are extracted from nonadiabatic molecular dynamics (NAMD) trajectories. The number of trajectories with a 0.5 fs time step (bright yellow) is as follows: **A01** (338), **A02** (408), **A03** (58), **R02** (118), and **I01** (200). Additionally, **R02** includes 18 trajectories with a 0.25 fs time step (dark yellow).

## Technical Validation

We provide datasheets for all nine molecules of the SHNITSEL dataset in the Supporting Information (*cf*. Figs. S1–18), offering visual representations of their multidimensional data. These datasheets quantify the diversity of quantum chemical properties by analyzing distributions of key quantities, such as state energies (*E*_*i*_) and the Frobenius norms of forces (**F**_*i*_), nonadiabatic couplings (**NAC**_*ij*_), transition dipole moments (*μ*_*ij*_), and spin-orbit couplings (**SOC**_*ij*_). Additionally, the datasheets of the trajectory data visualize correlations between these properties to provide further insight into the dataset characteristics.

To evaluate conformational diversity, principal component analysis (PCA) plots are provided for all molecules. For static datasets, PCA was performed using the Smooth Overlap of Atomic Position (SOAP) descriptor^81^, with structures projected onto the space defined by the three most significant principal components. For dynamic datasets, PCA was performed using molecular distance matrices as structural descriptors to identify and visualize geometrical clustering patterns arising from structural features such as interatomic distances or torsional angles. To capture temporal evolution, individual data points in the PCA projections were color-coded according to their corresponding simulation time.

For technical validation, we highlight representative visualizations for the herein, newly introduced data, *i.e*., the static data of **R01** and the surface hopping trajectory data of **A01–A03**.

### Static data: R01

**Fig. 4:**
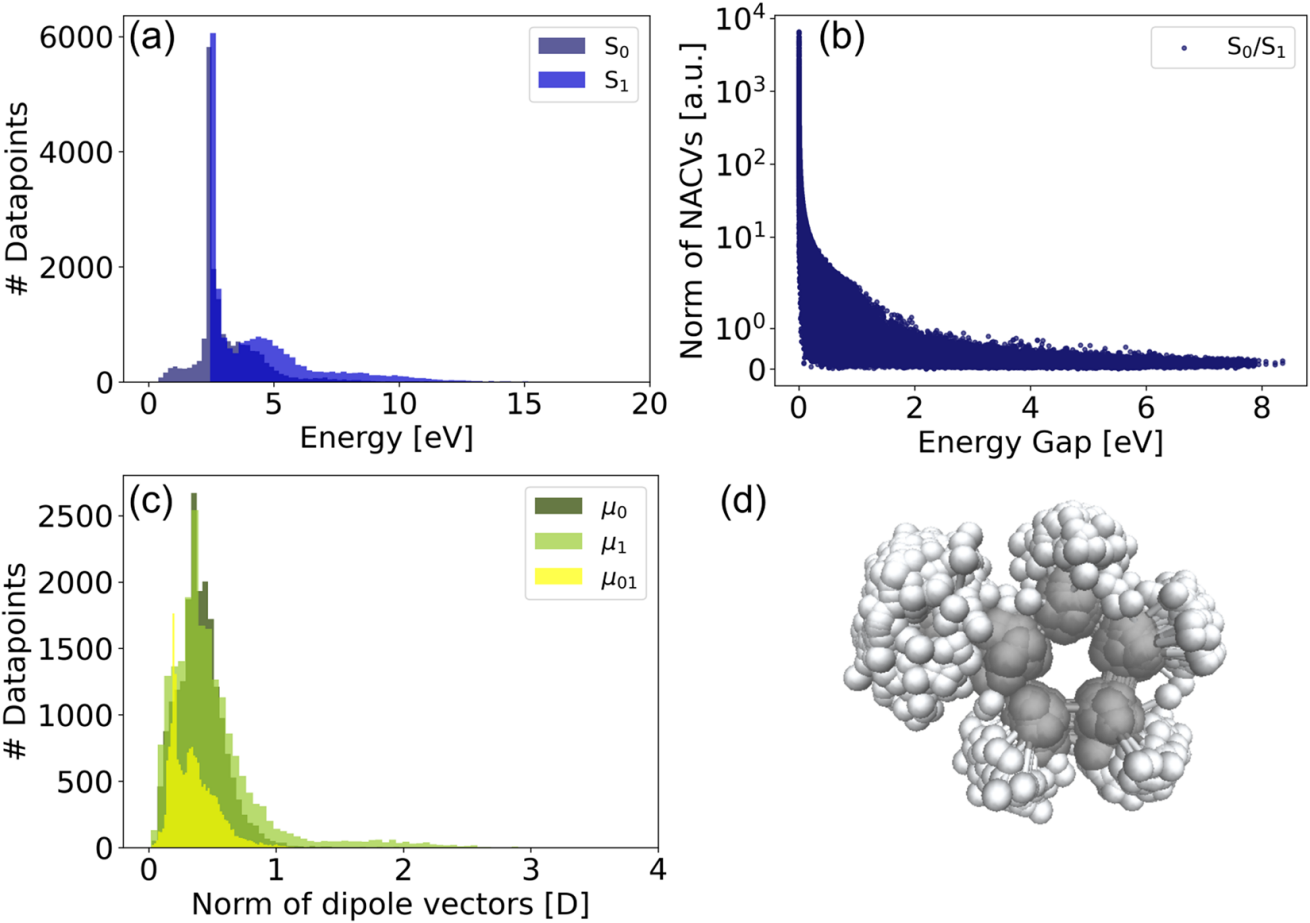
Distribution of (**a**) the energies, (*E*_*i*_), (**b**) norm of the NACs, (*NAC*_*ij*_), (**c**) the norm or the permanent and transition dipole moment vector (*μ*_*i*_ and *μ*_*ij*_) and (**d**) overlay of 3D-structure visualizations of all geometries included in the dataset of **R01**.

Figure 4 shows the properties in the dataset of **R01** with two singlet states being described. As can be seen from panel (a), the ground state energy ranges from 0 to about 5 eV, whereas most structures falling within an energy range of approximately 2 to 3 eV. Note that the equilibrium ground-state energy is set to 0 eV as a reference. Panel b) shows the distribution of the norms of the NACs versus the energy gap, showing increased NACs for lower energy gaps, as expected. Panel c) shows the distribution of the norm of the permanent and transition dipole moment vectors (*μ*_*i*_ and *μ*_*ij*_). The majority of values are distributed between 0 and 1 Debye. The distribution of the transition dipoles (*μ*_01_) shows a maximum at ≈0.1 Debye, which is lower than for the permanent dipoles in ${{\mathsf{S}}}_{{\mathsf{0}}}$ and ${{\mathsf{S}}}_{{\mathsf{1}}}$, where the maxima are found ≈0.5 Debye. Panel (d) visualizes the distribution of geometries in the dataset.

The PCA plot highlighting the three most important components, as obtained by means of PCA performed on all structures in the **R01** dataset using the SOAP^ 81^ descriptor is shown in Fig. S11. The PCA results confirm that no dissociated structures are present in the dataset of **R01**, which aligns with expectations^56^. Consequently, adaptive sampling did not explore dissociative regions. The distribution of transition energies ($| {{\mathsf{S}}}_{1}-{{\mathsf{S}}}_{0}|$) in the PCA plot further suggests that a broad conformational space was sampled during the four adaptive sampling runs.

### Dynamic data: A01–A03

Datasheets for surface hopping trajectory data of molecules **A01,** **A02** and **A03** are provided in the Supporting Information (see Figs. S4, S7 and S10), including *e.g*. the distribution of the potential energies, Frobenius norm of the forces and norm of the NACs as well as the correlation between properties (e.g. norm of the transition dipoles *vs*. energy gaps).

**Fig. 5:**
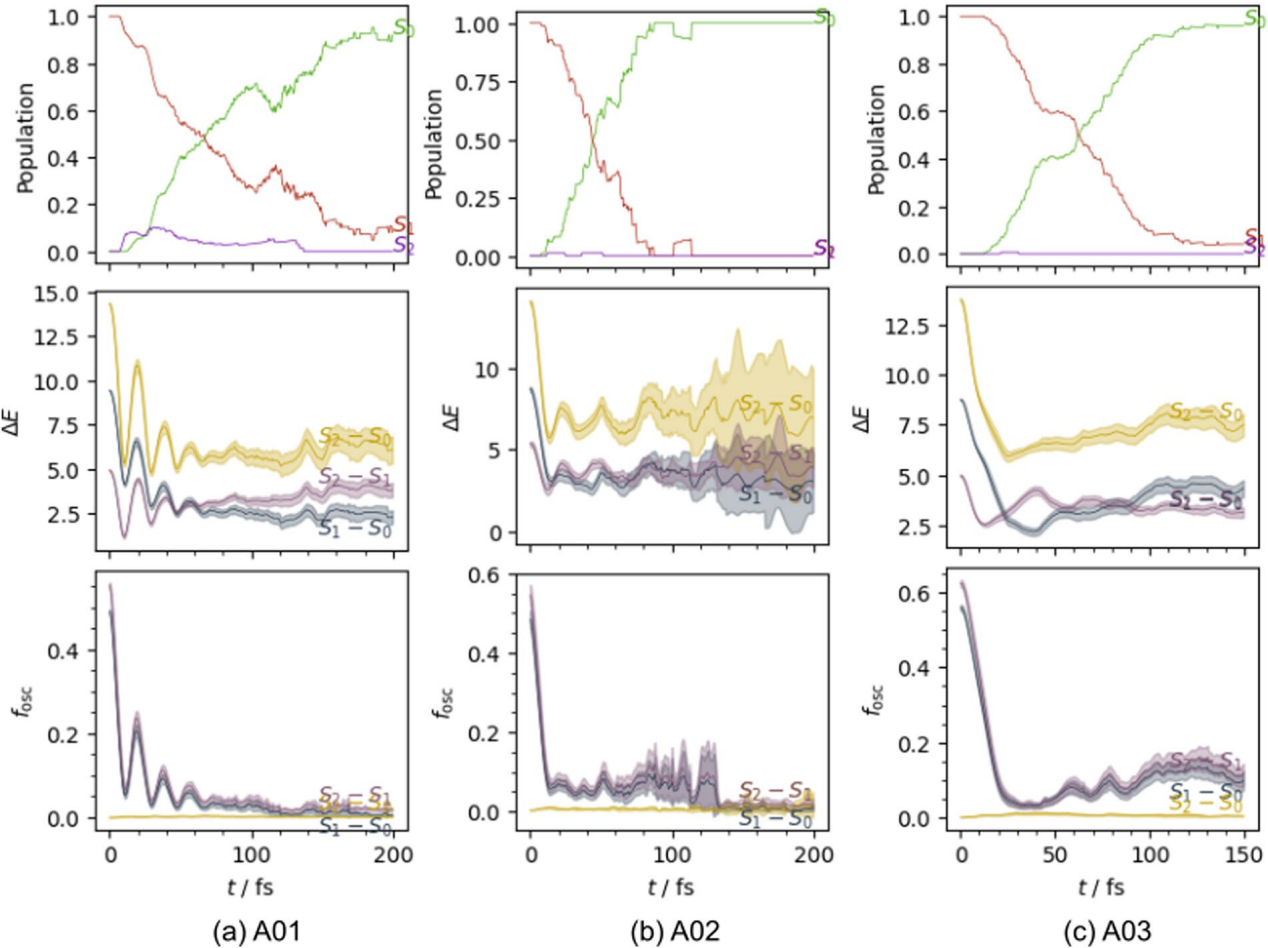
Visualization of the evolution of populations in the three lowest singlet states, along with the corresponding energy gaps and oscillator strengths for transitions between these states, for **A01,** **A02** and **A03**.

In Fig. 5 we show the normalized population curves for ${{\mathsf{S}}}_{{\mathsf{2}}}$, ${{\mathsf{S}}}_{{\mathsf{2}}}$, and ${{\mathsf{S}}}_{{\mathsf{3}}}$ (normalization of population of ${{\mathsf{S}}}_{{\mathsf{1}}}$ at *t*_0_ to 1), the change of the vertical excitation energies (middle row) and oscillator strengths (bottom row) between these states with simulation time for **A01,** **A02** and **A03**. These visualization reveals, that for **A03** the energy gap between ${{\mathsf{S}}}_{{\mathsf{1}}}$ and ${{\mathsf{S}}}_{{\mathsf{2}}}$ remains largely constant throughout the dynamics. In contrast, the gap between the ground state (${{\mathsf{S}}}_{{\mathsf{0}}}$) and the excited states (${{\mathsf{S}}}_{{\mathsf{1}}}$ and ${{\mathsf{S}}}_{{\mathsf{2}}}$) decreases markedly within the first 40 fs, suggesting that the system reaches the ${{\mathsf{C}}{\mathsf{I}}}_{10}$ conical intersection within this timeframe. This trend is mirrored in the behavior of the ${{\mathsf{S}}}_{1}\to {{\mathsf{S}}}_{2}$ excited-state absorption band, which remains spectrally stable (300–900 nm) but exhibits a pronounced decrease in intensity from an oscillator strength of approximately 0.6 to 0.05 within the first 40 fs. This decline reflects the rapid depopulation of the ${{\mathsf{S}}}_{{\mathsf{1}}}$ state and corresponds to an estimated ${{\mathsf{S}}}_{{\mathsf{1}}}$ lifetime of around 40 fs, as evidenced by the state population dynamics.

**Fig. 6:**
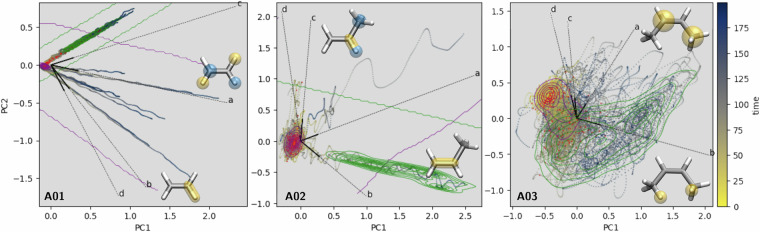
Two-dimensional principal component analysis (PCA) plot illustrating the configuration space distributions from the SHNITSEL dataset. The analysis is based on NAMD trajectories: 297 for **A01**, 84 for **A02**, and 87 for **A03**. PCA employed the distance matrix as a structural descriptor. Each symbol’s color corresponds to simulation time, with red indicating hopping geometries. Green-highlighted regions denote distinct photoproducts: C-H bond dissociation (**A01**, d(C-H)>200 pm), C=C bond dissociation (**A02**, d(C=C)>200 pm), and *Z* → *E* photoisomerization (**A03**), δ(HC=CH)<80°), as defined by internal coordinate constraints given in parenthesis. The four principal component vectors with the greatest variance (a > b > c > d) are shown, with inset structures highlighting the highest-contributing atom pairs for two selected vectors.

The PCA results obtained from the trajectory ensemble of the homologous series of the alkenes **A01,** **A02** and **A03** are shown in Fig. 6, where the dominant structural modes are projected into a reduced dimensionality space. Key atomic contributions to selected principal components are illustrated in the corresponding Markush representations of the molecules, highlighting atom pairs involved in significant structural rearrangements. For instance, in the case of **A03**, the PCA analysis emphasizes the critical role of the distance between the two outer hydrogen atoms, which helps differentiate the *Z*- and *E*-isomers of **A03** (see yellow highlighted atoms in the molecular structures shown as insets in Fig. 6). The respective kernel density estimation (KDE) surfaces highlight the *E*-isomer (green, 0°≤*δ*(HC=CH)<80°) and the *Z*-isomer (purple, 110°<*δ*(HC=CH)≤180°) on the PCA plot. This result aligns with the expected isomerization behavior, where the distance between the methyl groups increases during the transition between the *E*- and *Z*-geometries, enabling the identification of trajectories leading to the photoproduct (here: *Z*-but-2-ene → *E*-but-2-ene). Similarly, for the smaller alkene in the homologous series, **A01** and **A02**, the PCA plot reveals trajectories, where either the alkene bond or specific C-H bonds are stretched during the excited-state dynamics. These behaviors are visualized in clusters within the PCA plot and reflected in the molecular structure’s loadings (see highlighted atoms in the structure insets in Fig. 6).

## Usage Notes

The SHNITSEL dataset is provided for each molecule individually, with static and dynamic datasets available separately when applicable, all in NETCDF format on Zenodo^55^. A summary of the data labels and their corresponding shapes can be found in Table 4.

**Table 4:** Overview of variables and their corresponding shapes as stored in the SHNITSEL dataset for trajectory data.

quantity	symbol	variable	shape	default unit
positions	**R**	atXYZ	*a* × 3	Bohr
energy	*E*_*i*_	energy	*s* × 1	Hartree
forces	**F**_*i*_	forces	*s* × *a* × 3	Hartree/Bohr
permanent dipoles	*μ*_*i*_	dip_perm	*s* × 3	Debye
transition dipoles	*μ*_*ij*_	dip_trans	$$\left(\begin{array}{c}s\\ 2\end{array}\right)\times 3$$	a.u.
nacs	**NAC**_*ij*_	nacs	$$\left(\begin{array}{c}s\\ 2\end{array}\right)\times a\times 3$$	a.u.
phases	*p*_*i*_	phases	*s* × 3	–
active state		astate	1	–

The letters represent the number of atoms (*a*) and electronic singlet states (*s*), while × 3 indicates a spatial dimension. The variable column gives the variable name used in the NetCDF file.

## Supplementary information

Supplementary information

## Data Availability

The SHNITSEL dataset is available on Zenodo^55^, and interactive plots of the molecular structures and visualizations of key properties (for trajectory data) are compiled on the SHNITSEL webpage (https://shnitsel.github.io/molecules).
