# Supplementary material for: Surface Hopping Nested Instances Training Set for Excited-state Learning
Source: Sci Data. 2025 Jul 26;12:1300. doi: 10.1038/s41597-025-05443-5 (PMC12297575; doi:10.1038/s41597-025-05443-5)
Supplement: Supplementary file 1 — Supplementary information [file 41597_2025_5443_MOESM1_ESM.pdf]

Supporting Information

Surface Hopping Nested Instances Training Set for Excited State Learning

Robin Curth[1,2,†], Theodor E. Röhrkasten[3,†], Carolin Müller[3,*], Julia Westermayr[1,2,*]

[1] Leipzig University, Wilhelm Ostwald Institute for Physical and Theoretical Chemistry, Linnéstraße 2, Leipzig, 04103, Germany
[2] Center for Scalable Data Analytics and Artificial Intelligence (ScaDS.AI), Dresden/Leipzig, Humboldtstraße 25, Leipzig, 04105, Germany
[3] Friedrich-Alexander-Universität Erlangen-Nürnberg, Computer-Chemistry-Center, Nägelsbachstraße 25, Erlangen, 91052, Germany

[*] correspondence: julia.westermayr@uni-leipzig.de, carolin.cpc.mueller@fau.de
[†] These authors contributed equally to this work.

Contents

1 Datasheets **2**
1.1 **A01** . . . 2
1.2 **A02** . . . 7
1.3 **A03** . . . 10
1.4 **R01** . . . 13
1.5 **R02** . . . 14
1.6 **R03** . . . 16
1.7 **I01** . . . 18
1.8 **H01** . . . 21
1.9 **T01** . . . 23

1 Datasheets

1.1 A01

The distributions of energies and excited-state properties for the three datasets of **A01** are presented in Figures S1, S2, and S3. The energy and coupling distributions closely resemble those of the isoelectronic molecule **I01** (see FigureS15). For **A01**, the majority of permanent dipole moments in both the ground and excited states fall within the range of 1 to 8 Debye. The transition dipole moment between the ground state and the first excited singlet state, $\boldsymbol{\mu}_{01}$, is notably large, contributing to the intense absorption band associated with the $\mathsf{S}_0 \rightarrow \mathsf{S}_1$ excitation. [1–4]

Ethene a (C_2H_4)

Database Name	A01_ethene_a_static.nc
# Datapoints	3,969
Reference Method	MR-CISD/aug-cc-pVDZ (SA(3)-CASSCF(6,4))
States	3 Singlet
Properties	Energies, Forces, (Transition)dipoles, NACs

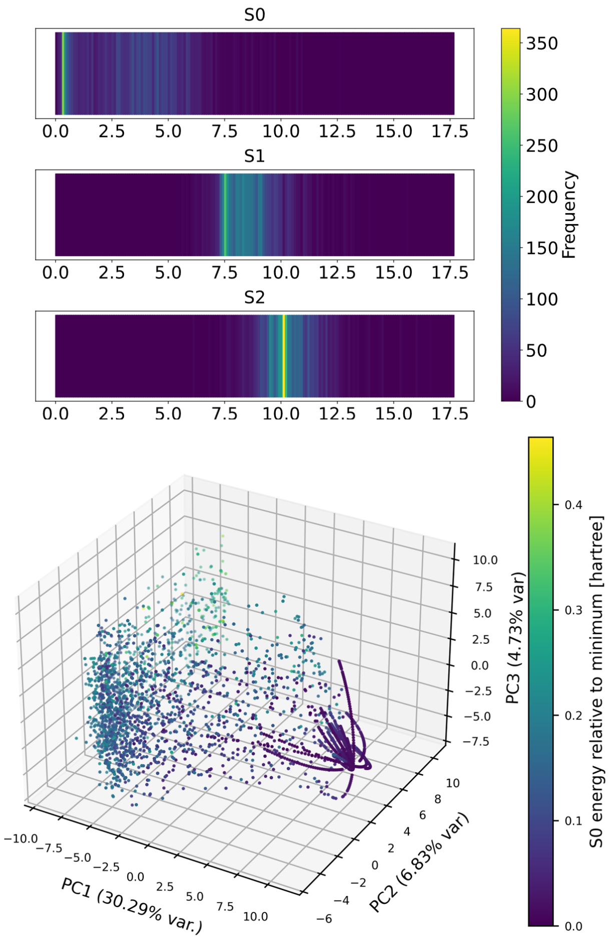

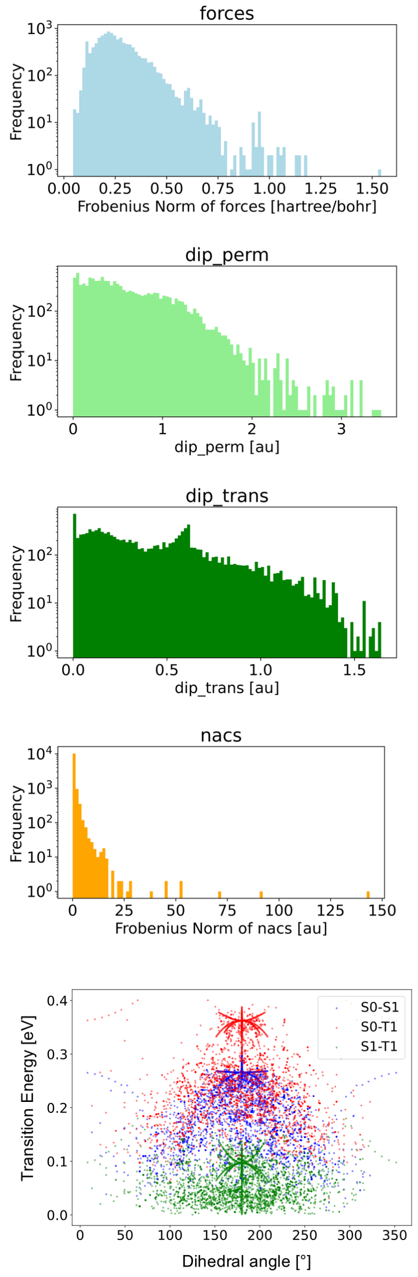

Figure S1: Overview of static data of **A01** (a).

Ethene b (C_2H_4)

Database Name	A01_ethene_b_static.nc
# Datapoints	5,999
Reference Method	MR-CISD/aug-cc-pVDZ (SA(3)-CASSCF(2,2))
States	3 Singlet
Properties	Energies, Forces, (Transition)dipoles, NACs

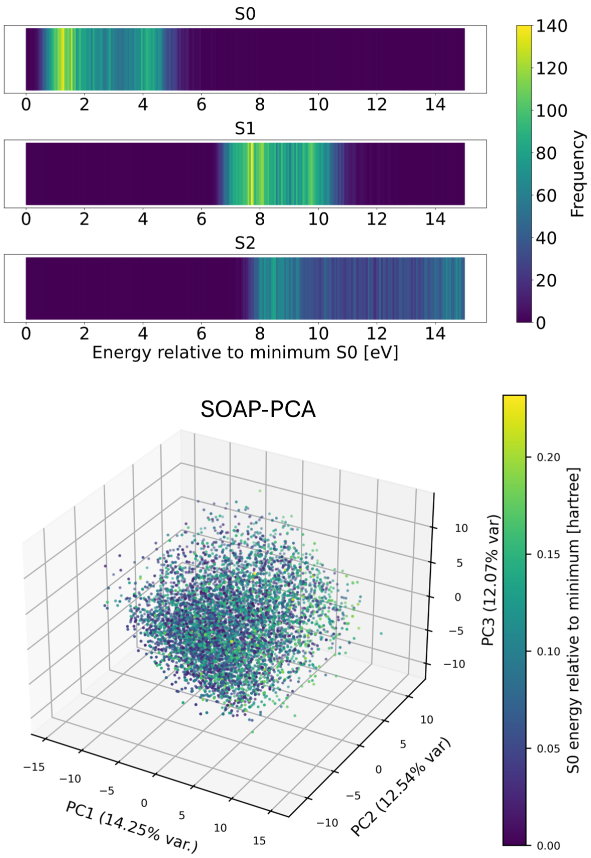

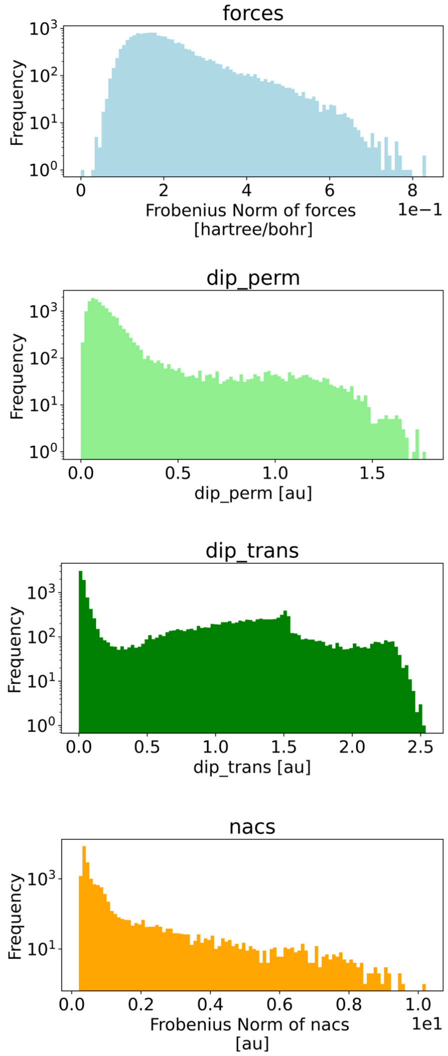

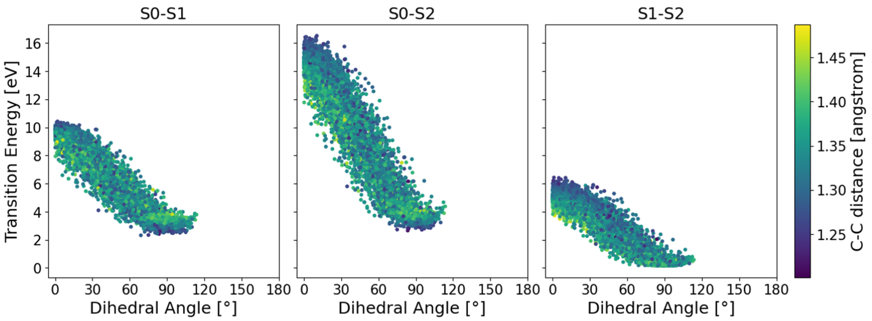

Figure S2: Overview of static data of **A01** (b).

Ethene Grid (C_2H_4)

Database Name	A01_ethene_grid_static.nc
# Datapoints	3,731
Reference Method	MR-CISD/aug-cc-pVDZ (SA(3)-CASSCF(2,2))
States	3 Singlet
Properties	Energies, Forces, (Transition)dipoles, NACs

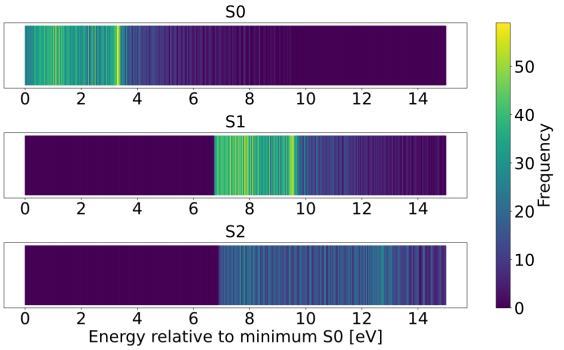

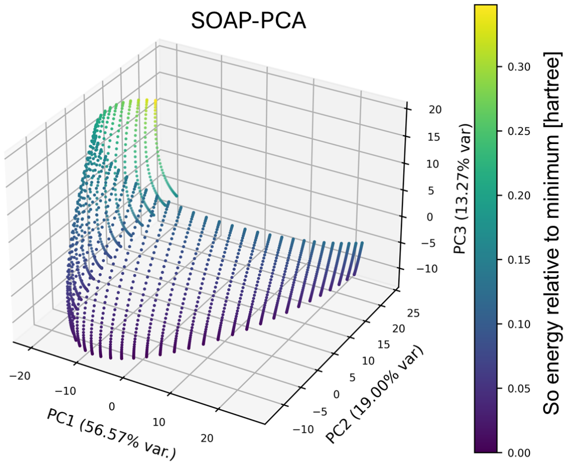

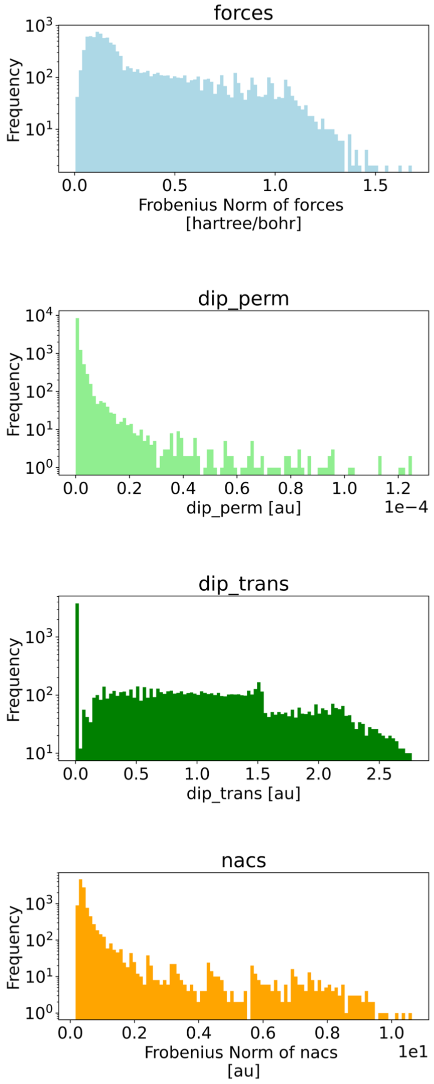

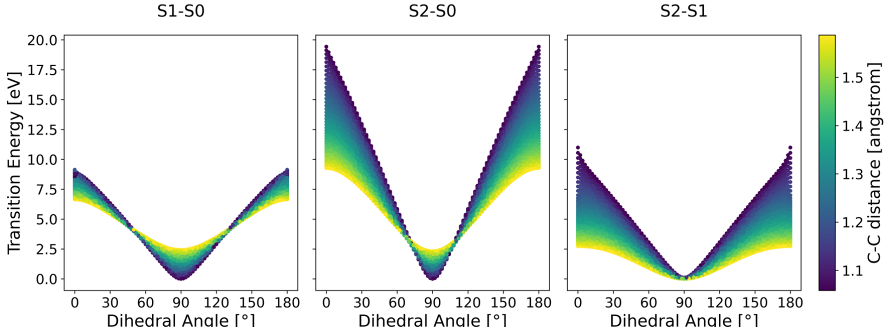

Figure S3: Overview of static data of **A01** (grid).

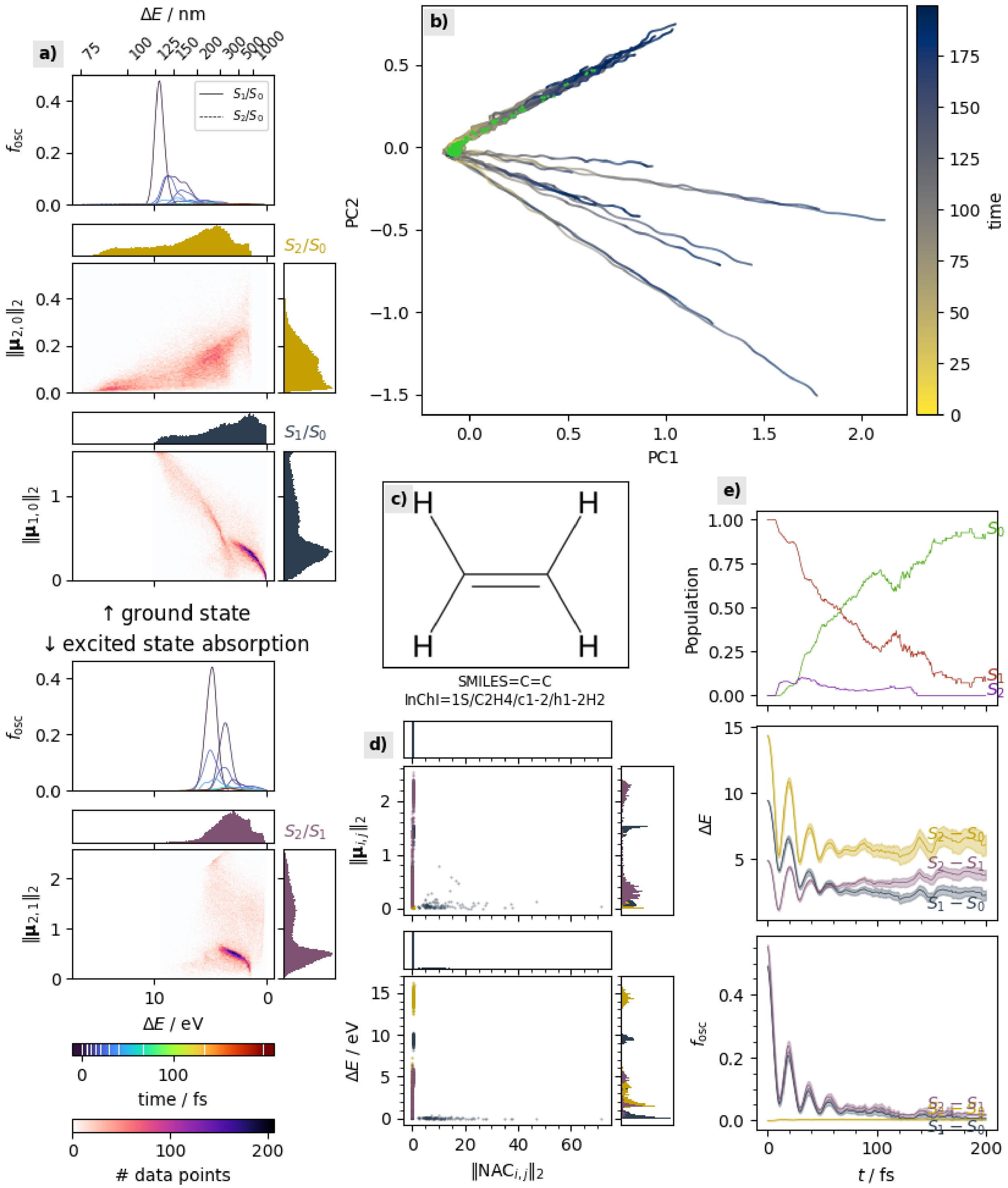

Figure S4: Overview of trajectory data of **A01** (C_2H_4, ethene), 62,031 data points, data averaged over 297 trajectories ($\Delta t = 0.5$ fs).

1.2 A02

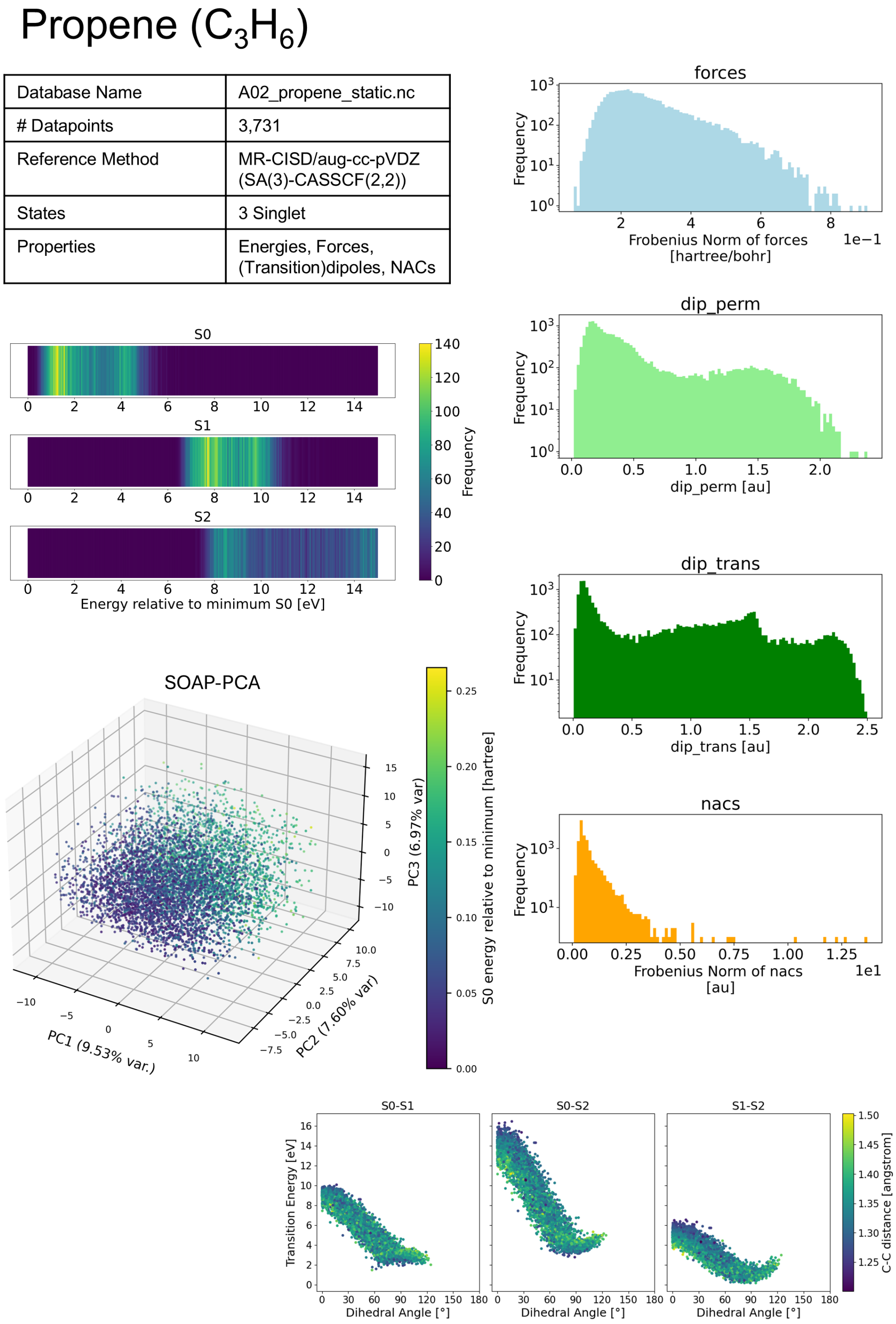

Figure S5: Overview of static data of **A02** (a).

Propene Grid (C_3H_6)

Database Name	A02_propene_grid_static.nc
# Datapoints	3,731
Reference Method	MR-CISD/aug-cc-pVDZ (SA(3)-CASSCF(2,2))
States	3 Singlet
Properties	Energies, Forces, (Transition)dipoles, NACs

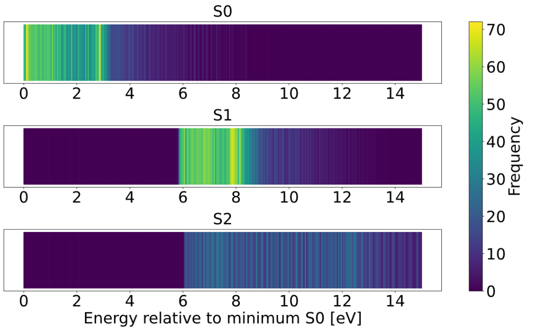

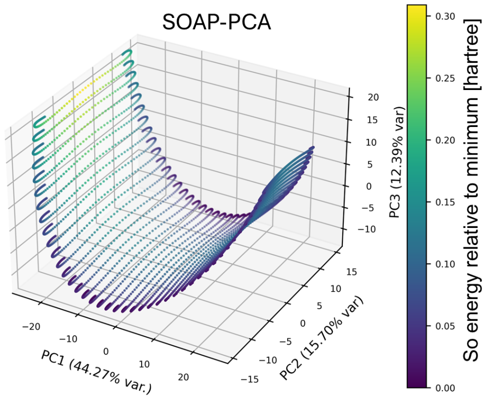

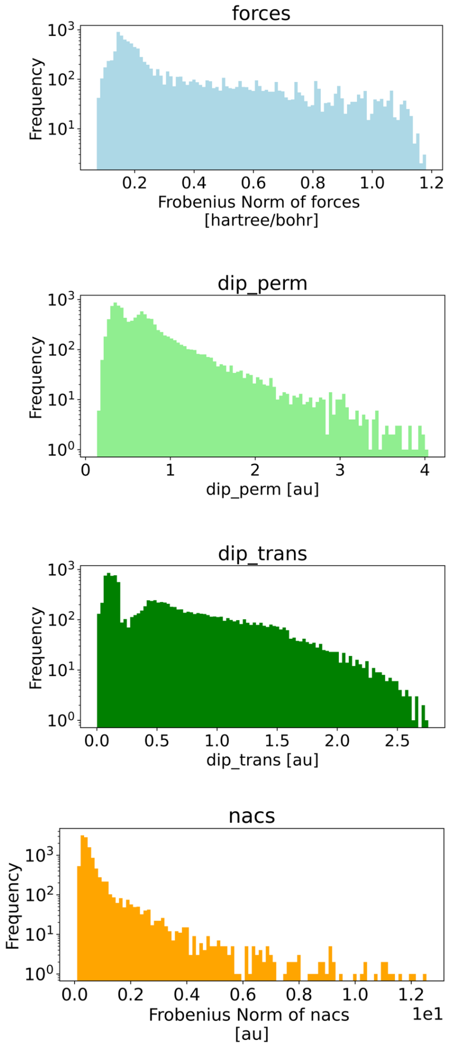

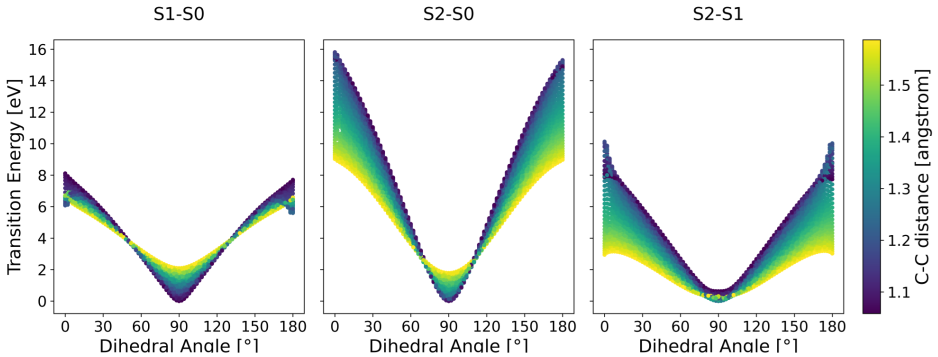

Figure S6: Overview of static data of **A02** (grid).

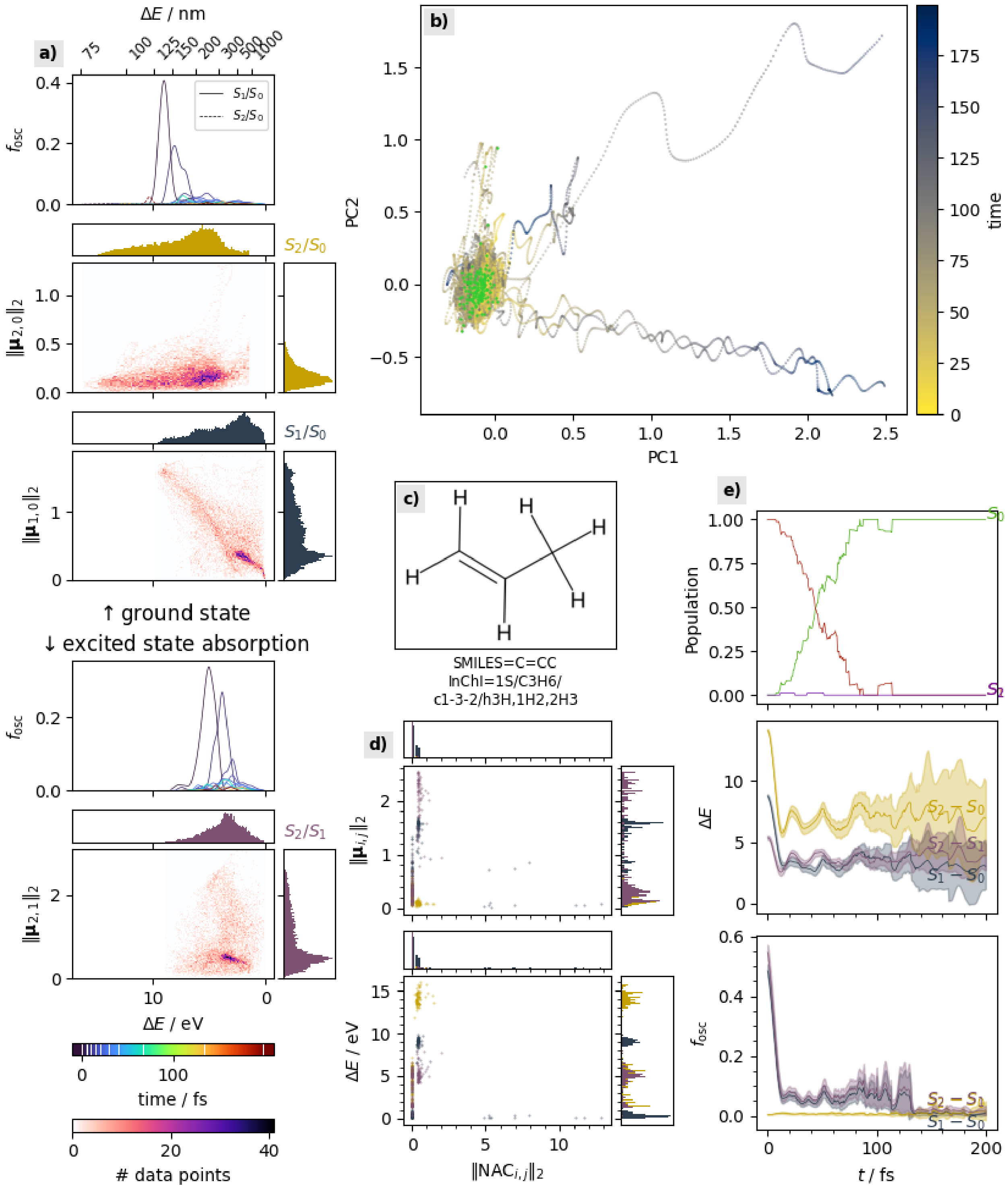

Figure S7: Overview of trajectory data of **A02** (C_3H_6, propene), 13,899 data points, data averaged over 84 trajectories ($\Delta t = 0.5$ fs).

1.3 A03

Butene (C_4H_8)

Database Name	A03_butene_static.nc
# Datapoints	3,969
Reference Method	MR-CISD/aug-cc-pVDZ (SA(3)-CASSCF(2,2))
States	3 Singlet
Properties	Energies, Forces, (Transition)dipoles, NACs

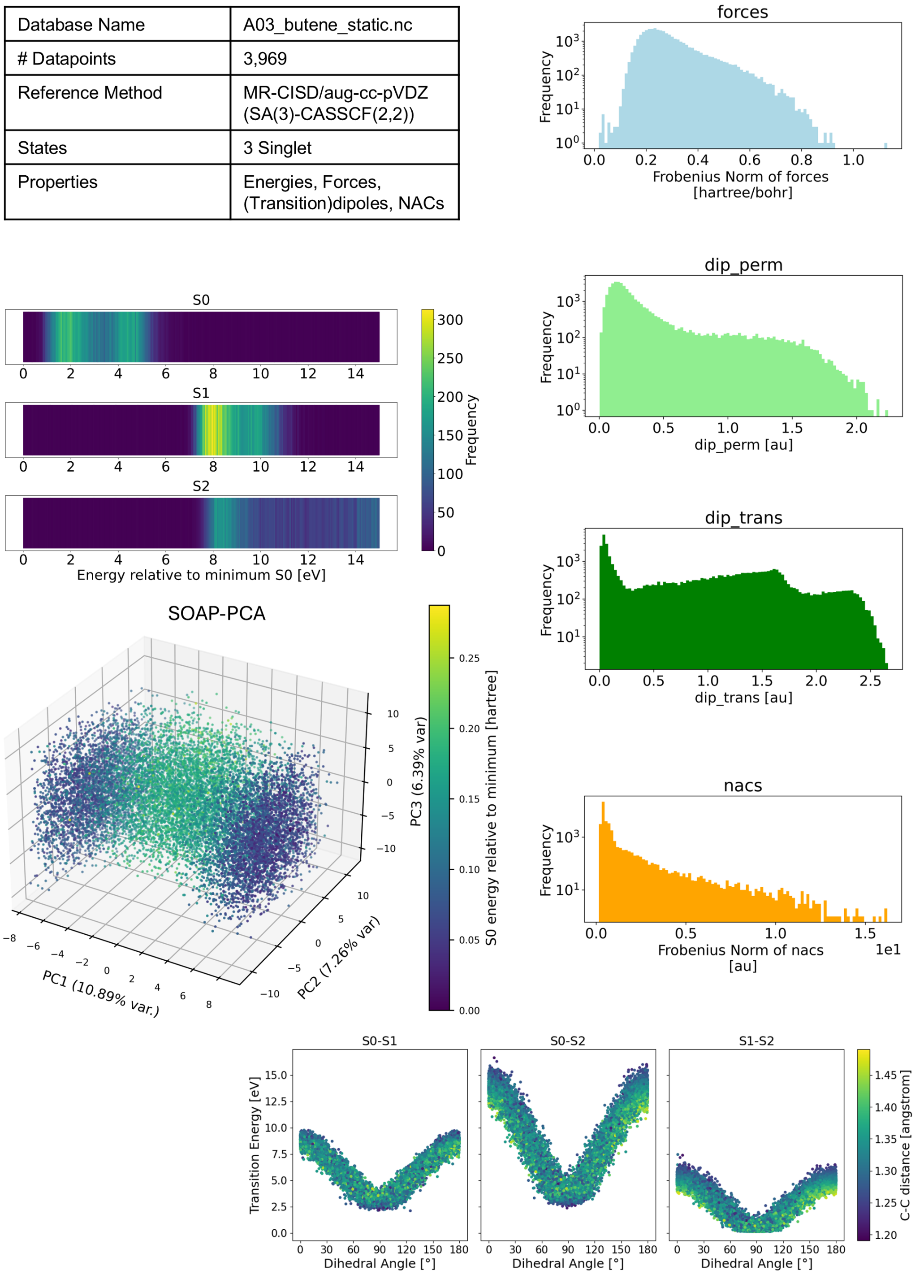

Figure S8: Overview of static data of **A03** (a).

Butene Grid (C_4H_8)

Database Name	A03_butene_grid_static.nc
# Datapoints	3,731
Reference Method	MR-CISD/aug-cc-pVDZ (SA(3)-CASSCF(2,2))
States	3 Singlet
Properties	Energies, Forces, (Transition)dipoles, NACs

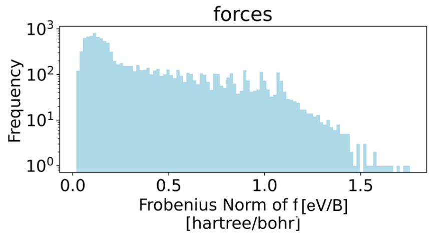

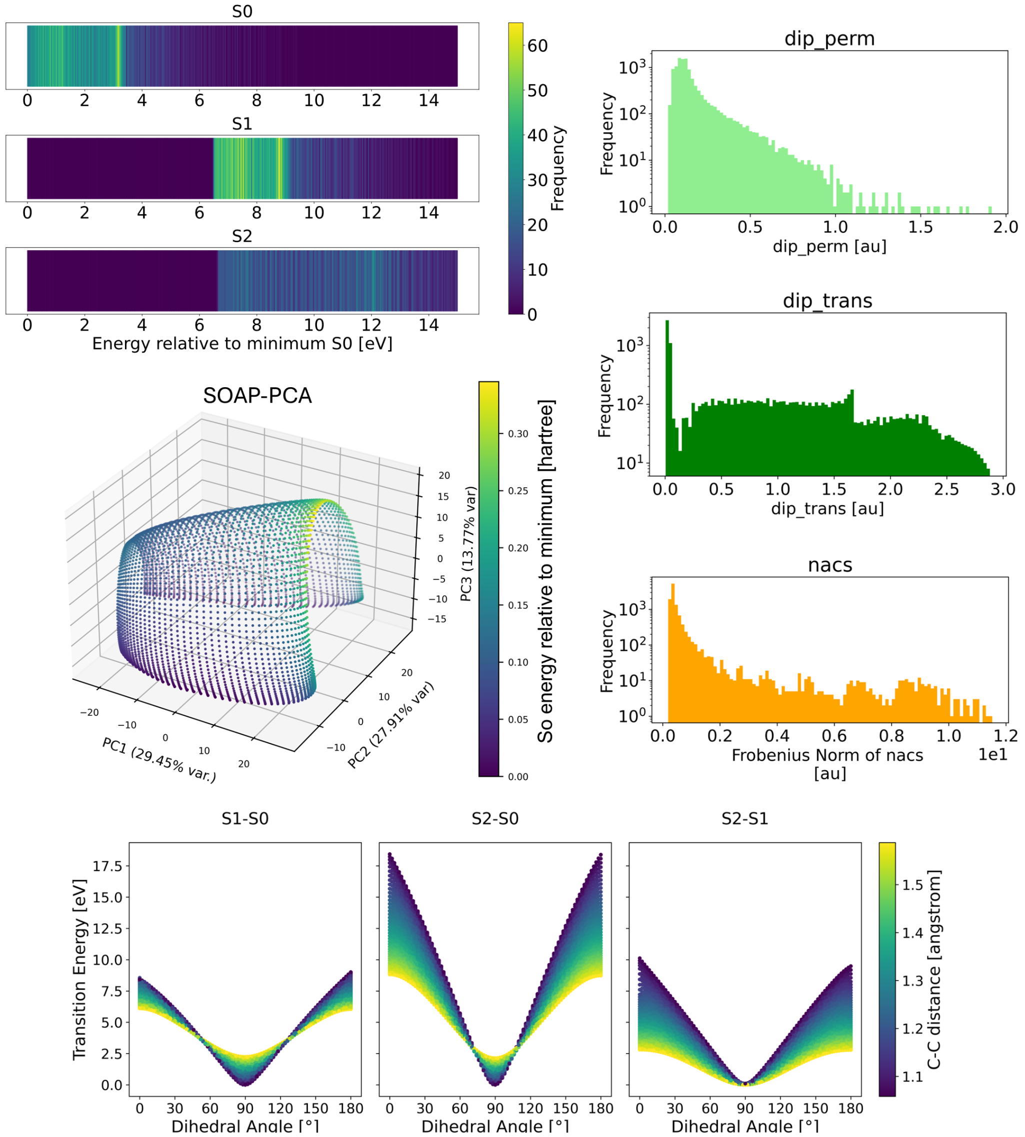

Figure S9: Overview of static data of **A03** (grid).

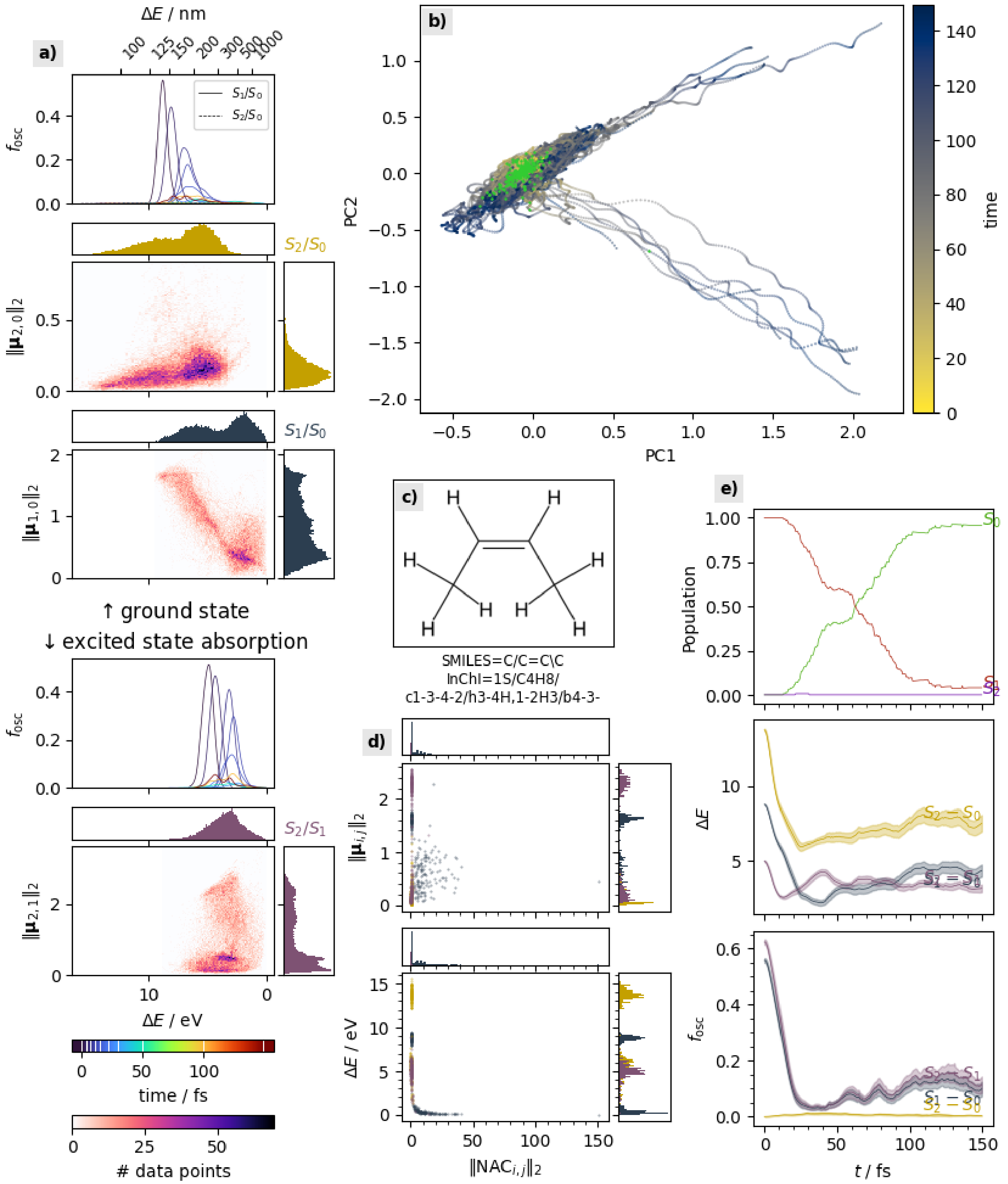

Figure S10: Overview of trajectory data of **A03** (C_4H_8, Z-but-2-ene), 36,579 data points, data averaged over 156 trajectories ($\Delta t = 0.5$ fs).

1.4 R01

Fulvene (C_6H_6)

Database Name	R01_fulvene_static.nc
# Datapoints	20,032
Reference Method	SA(2)-CASSCF(6,6)/ 6-31G*
States	2 Singlet
Properties	Energies, Forces, (Transition)dipoles, NACs

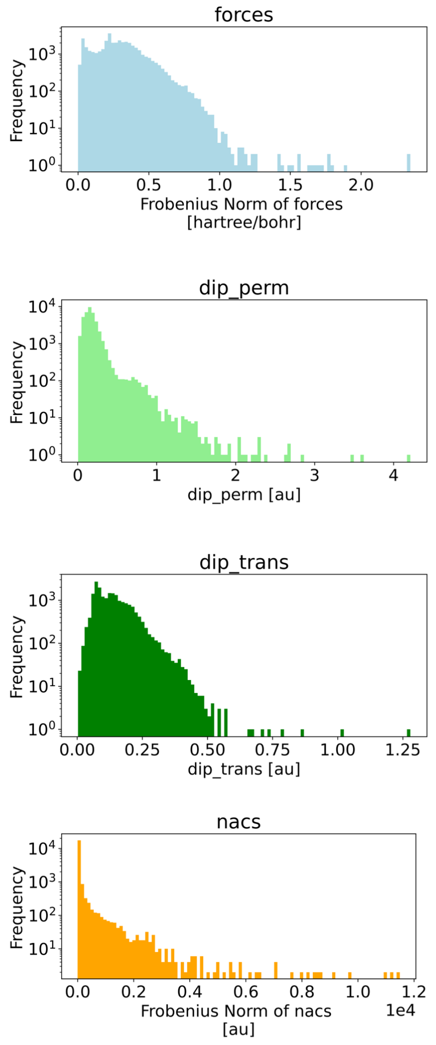

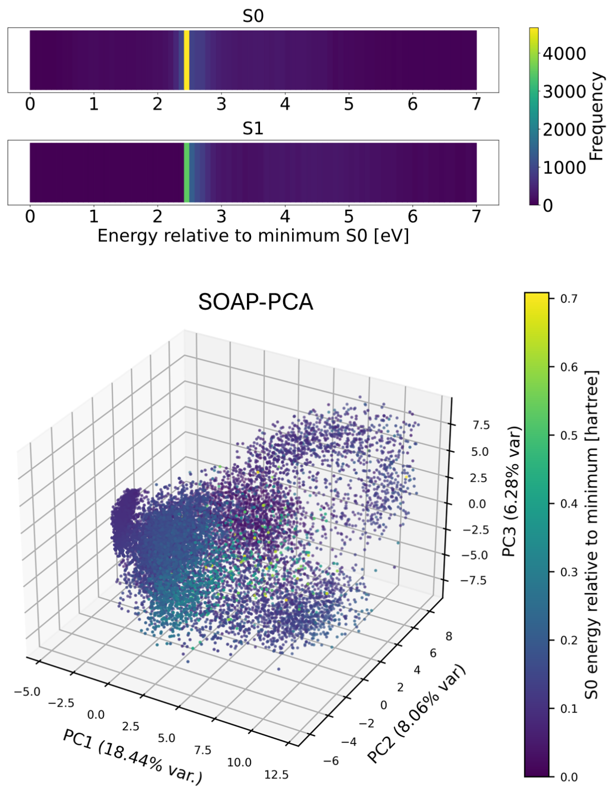

Figure S11: Overview of static data of **R01**.

1.5 R02

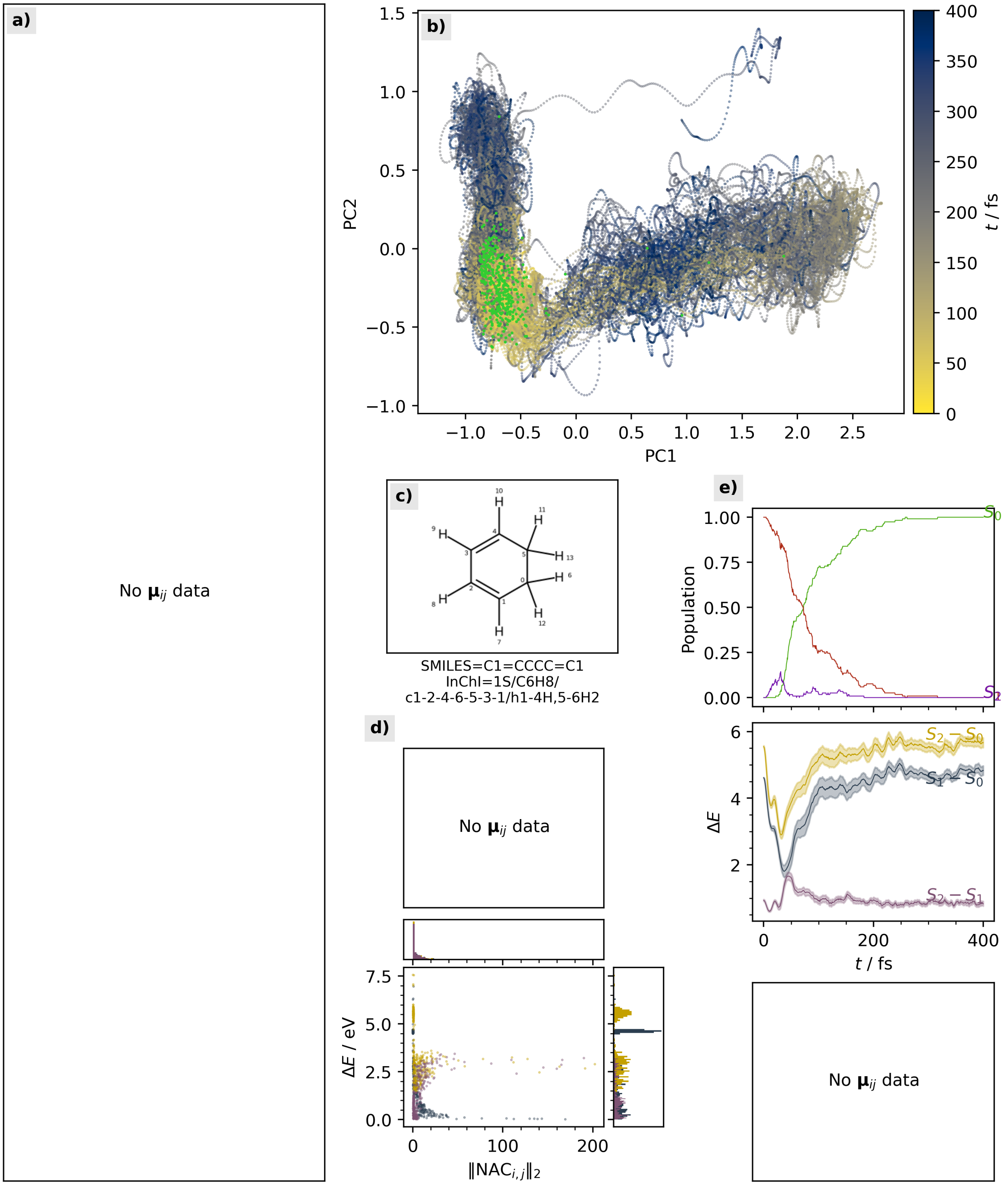

Figure S12: Overview of trajectory data of **R02**(a) (C_6H_8, 1,3-cyclohexadiene), 92,808 data points, data averaged over 118 trajectories ($\Delta t = 0.5$ fs).

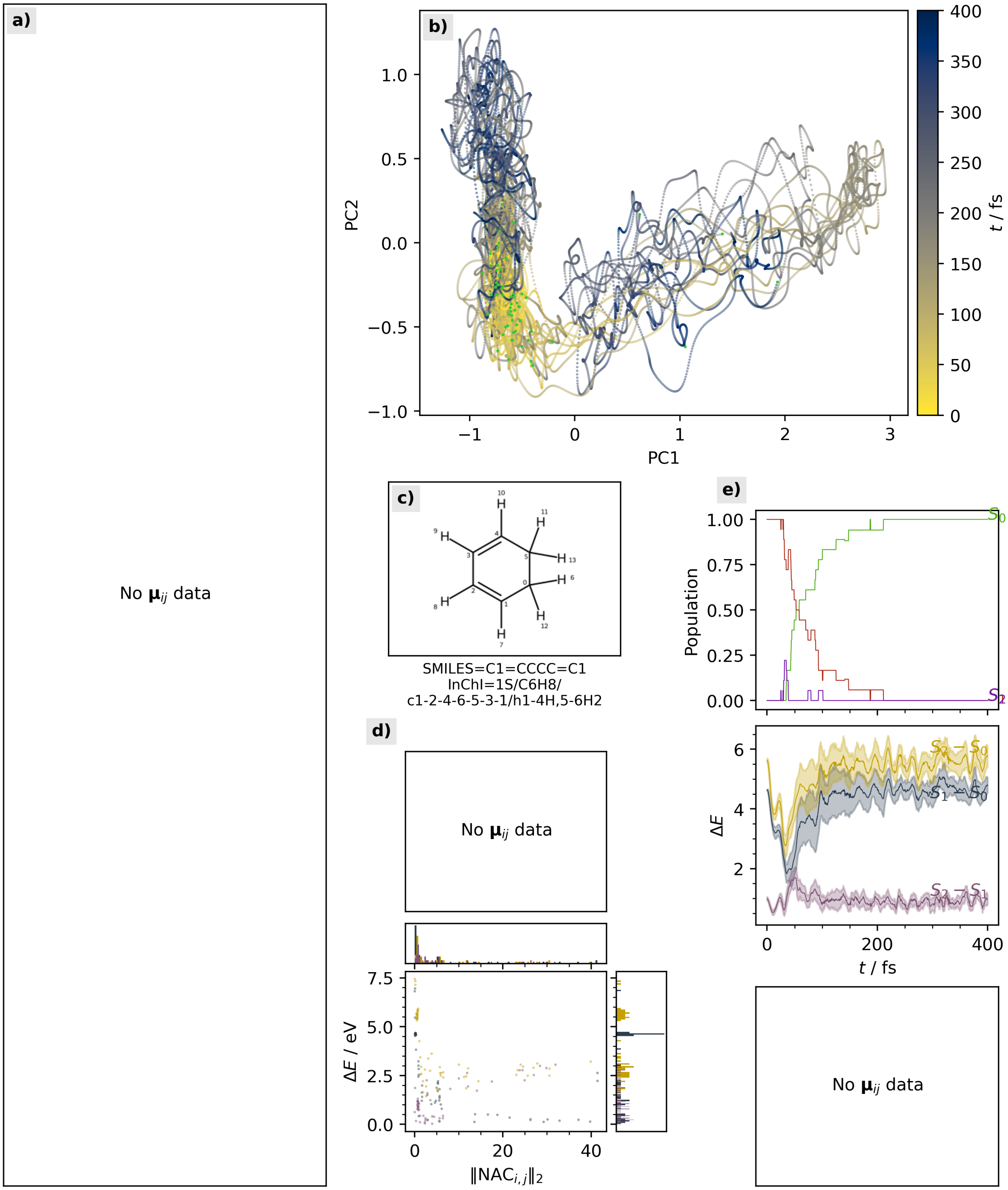

Figure S13: Overview of trajectory data of **R02**(b) (C_6H_8, 1,3-cyclohexadiene), 27,018 data points, data averaged over 18 trajectories ($\Delta t = 0.25$ fs).

1.6 R03

The amino acid tyrosine, **R03**, is the largest molecule described in the repository and includes singlet and triplet states. It is the first biologically relevant system in which roaming atoms have been discovered.[5] Due to the relatively large size of the system and at least 5 singlet states that have to be fitted, the learning of the photochemistry of this system is, similar to diiodomethane, not straightforward and can serve exciting new method developments. Recent studies have shown that only singlet states are relevant for the photodynamics simulations.[5, 6]

The distributions of singlet states and triplet states in the data set of tyrosine are shown on th left in Figure S14 with the molecular distribution being part of this panel. As can be seen, most values are located within 0 and 10 eV. However, there are also a few unfavourable structures in the data set. In addition, some data points with dissociated hydrogen atoms with distances up to 100 Å with respect to the originally attached carbon, oxygen, or nitrogen atoms.

On the right side the distribution of the norms of the permanent and transition dipole moment vectors are shown. Again, the dipole moment vectors of the same spin multiplicities are summarized. As in the case of diiodomethane, there are a few values with very large dipole moment vectors (see inset). On average, permanent dipole moment vectors are larger than transition dipole moment vectors.

The SOCs are shown and it is visible that SOCs are centred around 0 cm^{-1} with largest values up to ±65 cm^{-1}. The generally small SOCs indicate that intersystem crossing is not relevant in the system and that triplet states can be neglected in photodynamics simulations.

Tyrosine ($C_9H_{11}NO_3$)

Database Name	R03_tyrosine_static.nc
# Datapoints	17,265
Reference Method	ADC(2)/cc-pVDZ + CASPT2(12,11) / Ano-rcc-pVDZ
States	5 Singlet, 8 Triplet
Properties	Energies, Forces, (Transition)dipoles, SOCs

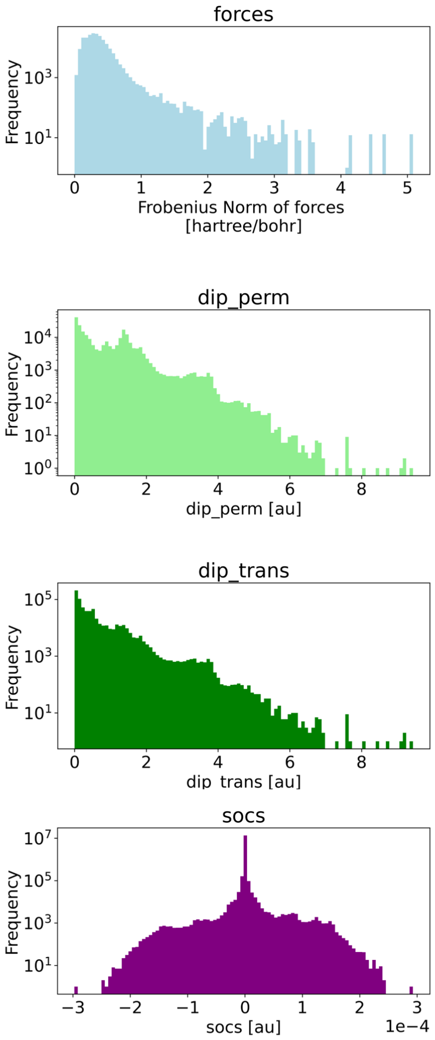

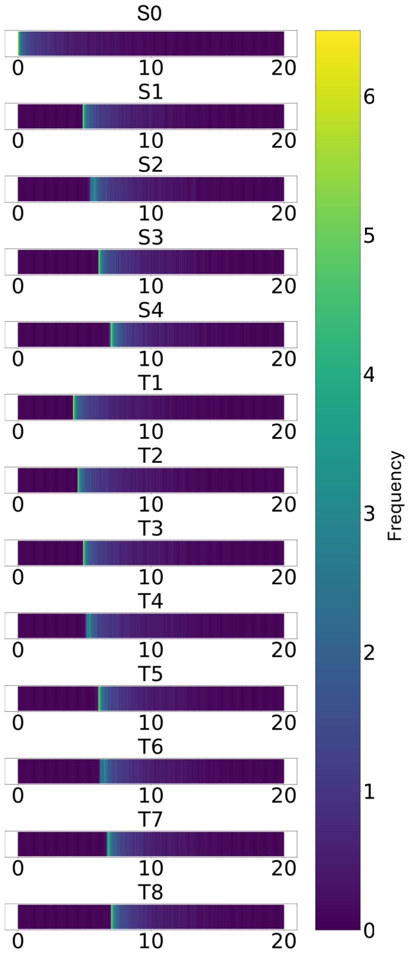

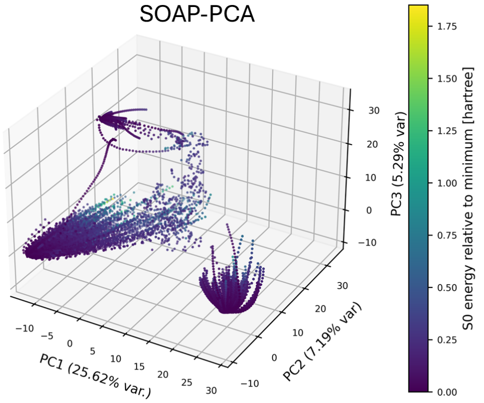

Figure S14: Overview of static data of **R03**.

1.7 I01

Figure S15 shows the properties in the data set of the **I01**. As can be seen, three singlet states are described. The ground state energy ranges from 0 to about 10 eV, whereas a great portion of data points lies close to the equilibrium structure. In this plot, and in all subsequent energy plots, the ground state energy of the equilibrium structure is set to 0 eV and all energies are shifted accordingly. Those data points refer to the initially sampled data points, where scans along the different normal modes were carried out to accurately sample the most relevant conformational regions for subsequent NAMD. The energy range of the excited states is narrower compared to the ground state.

Methylammonium Cation (CH_2NH_2+)

Database Name	I01_ch2nh2_static.nc
# Datapoints	4,000
Reference Method	MR-CISD/aug-cc-pVDZ (SA(3)-CASSCF(6,4))
States	3 Singlet
Properties	Energies, Forces, (Transition)dipoles, NACs

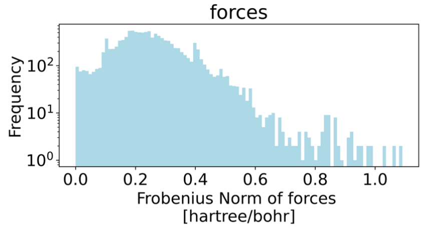

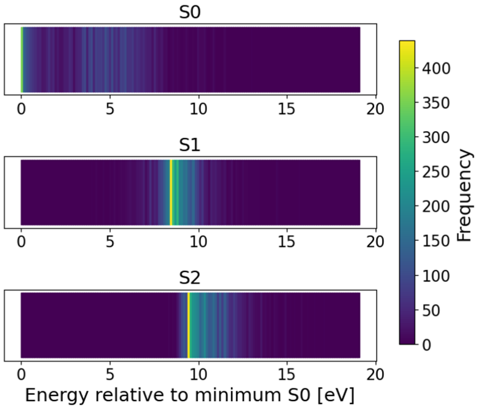

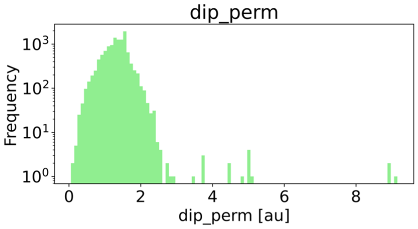

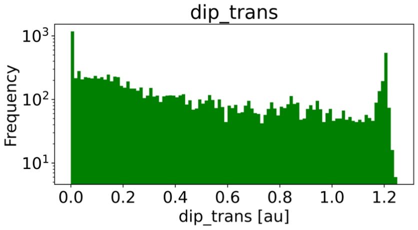

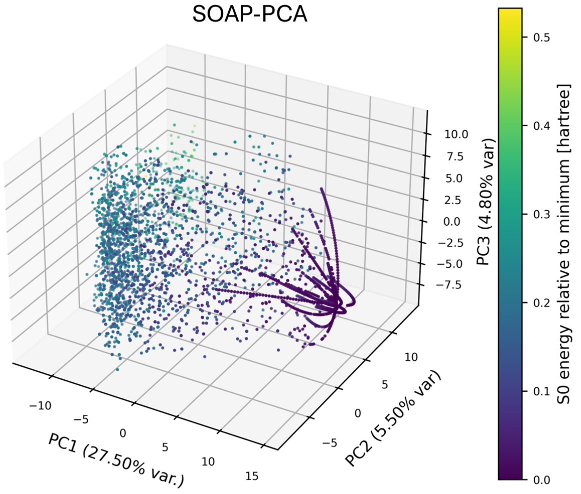

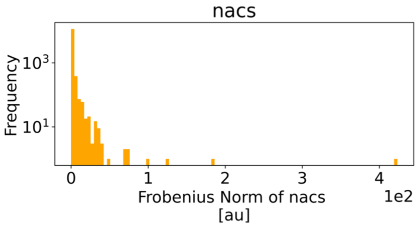

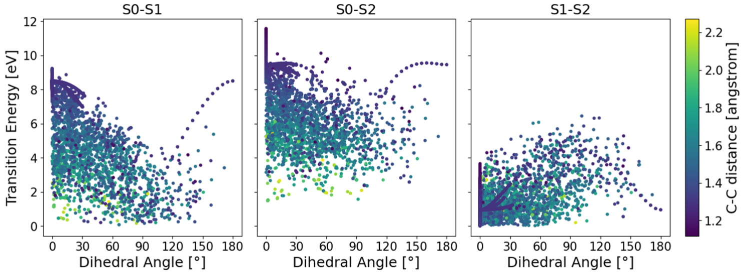

Figure S15: Overview of static data of **I01**.

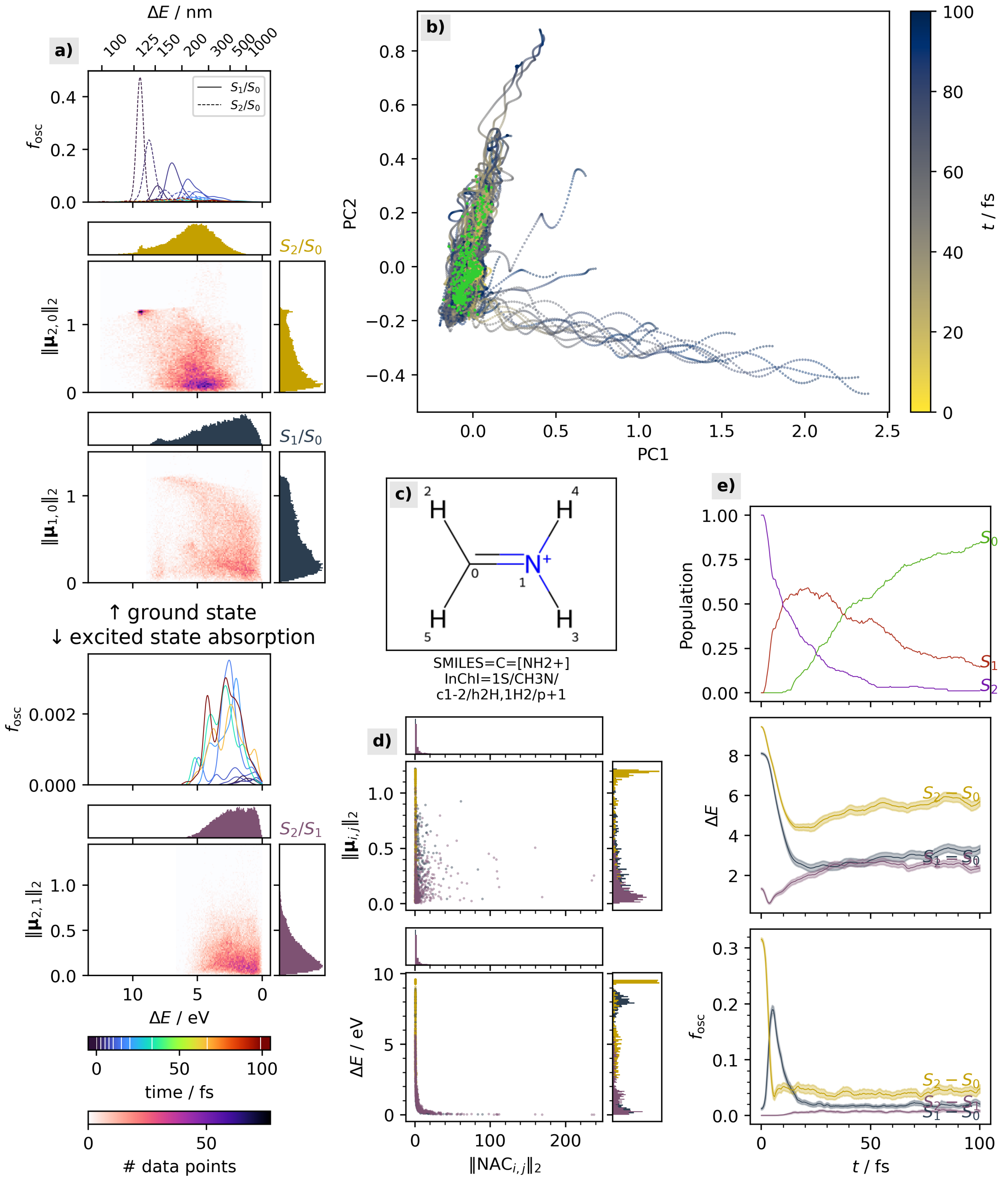

Figure S16: Overview of trajectory data of **I01** (CH_3N, methylene immonium), 39,365 data points, data averaged over 200 trajectories ($\Delta t = 0.5$ fs).

1.8 H01

Diiodomethane, **H01**, has strong spin-orbit couplings and the simulation of accurate photodynamics requires the consideration of several excited states (5 singlets and 4 triplets, which are 17 spin-mixed states). The fitting of that many excited states is especially challenging with ML techniques and this data set could serve the benchmarking of available methods and the development of new methods.

The data points for the training set of diiodomethane were obtained from surface hopping NAMD simulations of reference, [7] which already cover the relevant conformational space required for accurate photodynamics. For details on the trajectories, the reader is refered to ref. [7]. As dissociation of iodine is observed, calculations were computed at the CASPT2(12,8)/ano-rcc-vdzp level of theory, which only allowed for computationally expensive numerical force calculations. [7] In this way, we ended up with 60,856 data points. The large number of accurate multi-reference data points with 5 singlet and 4 triplet states fosters the development of novel ML models and cluster algorithms to find the optimal training set size and learning algorithm to fit many excited state energies and properties simultaneously. In CASPT2 calculations, the IPEA shift was set to 0. [7]

The energy distributions of the singlet states and triplet states of diiodomethane are shown in Fig. 18. The triplets are higher in energy than the singlets.

The norm of the permanent dipole moment vectors of all singlets and triplets are combined. Most norms of dipole moment vectors are within 0 and 4 Debye. However, there are some very large norms of permanent dipole moments. These large values are related to structures in which an iodine atom is dissociated. The large values are reasonable considering that the dipole moment vector is proportional to the distances of an atom to the center of mass of the molecule.

The distribution of SOCs is also shown. In contrast to **T01**, **H01** exhibits much stronger SOCs with absolute values being larger than 2000 cm^{-1}. This behaviour is in agreement with recent studies that found relatively large intersystem crossing rates. [7]

Diiodomethane (CH_2I_2)

Database Name	H01_diiodomethane_static.nc
# Datapoints	60,856
Reference Method	SA(5/4)-CASPT2(12,8)/ano-rcc-vdzp
States	5 Singlet, 4 Triplet
Properties	Energies, Forces, (Transition)dipoles, SOCs

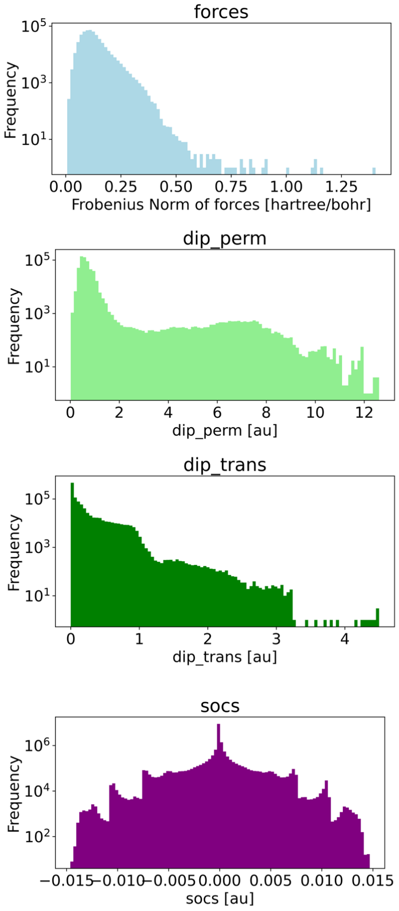

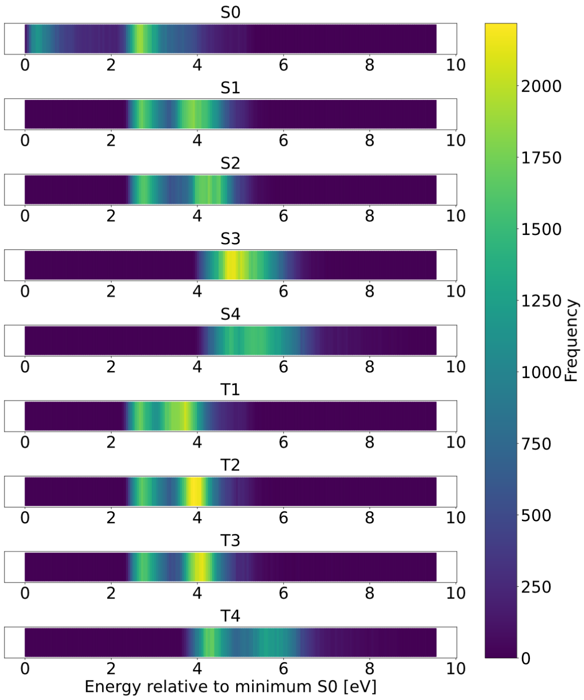

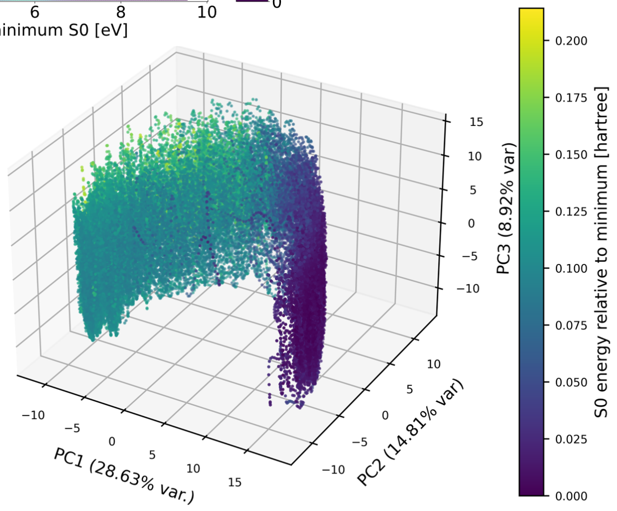

Figure S17: Overview of static data of **H01**.

1.9 T01

T01 has slow population transfer from the first excited state to the ground state and to triplet states, hence showing little intersystem crossing. [8] This is different to the previously mentioned molecules that are characterized by fast population transfer.

Fig. 18 shows the datasheet of **T01** including the distribution of the singlet states and triplet states. It is important that triplet states are within the energy range of the described singlet states or exceed this energy range. Due to triplet states, SOCs are included in the calculations. Most SOC values are very close to 0. However, there are also comparably few large values which are important in the system. The norm of the permanent dipole vectors of the first and second triplet states is smaller than those of the ground state and the first excited singlet state, respectively.

Methanethione (CSH_2)

Database Name	T01_methanethione_static.nc
# Datapoints	4,855
Reference Method	SA(2/2)-CASSCF(6,5)/ def2SVP
States	2 Singlet, 2 Triplet
Properties	Energies, Forces, (Transition)dipoles, SOCs

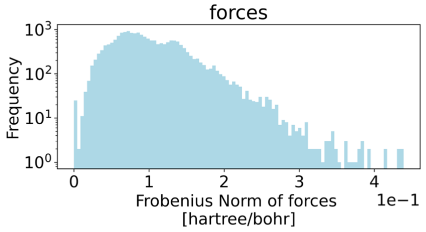

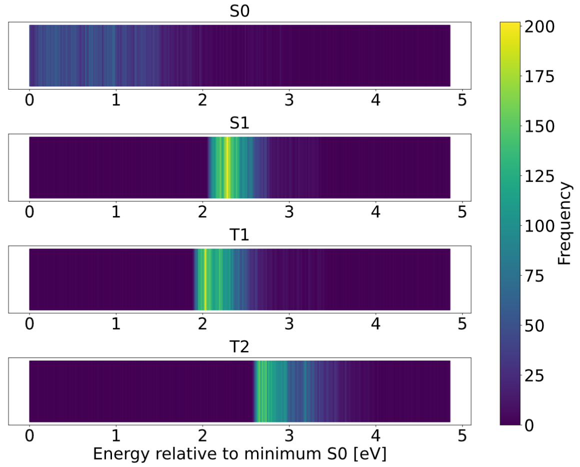

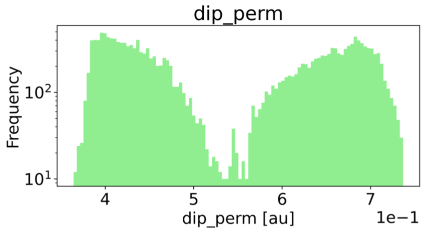

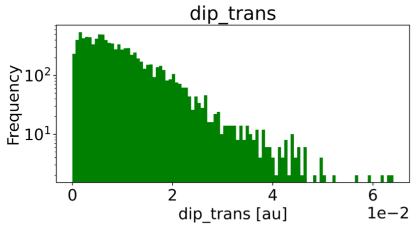

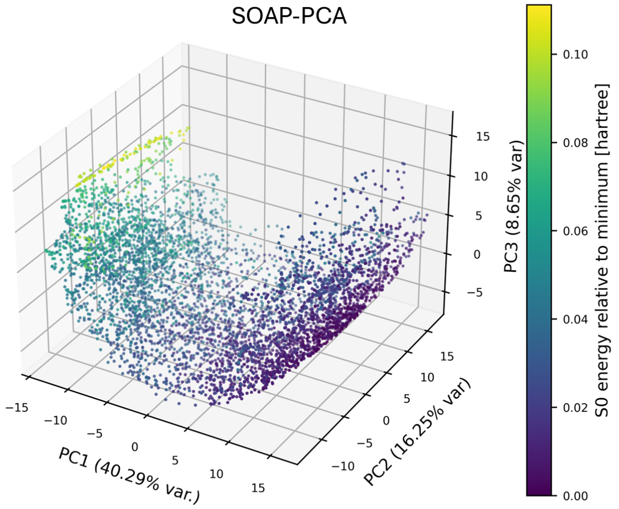

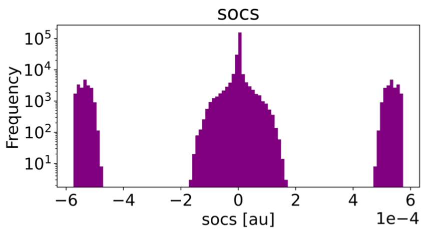

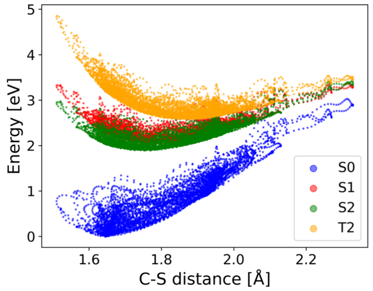

Figure S18: Overview of static data of **T01**.

References

1. Barbatti, M., Aquino, A. J. A. & Lischka, H. Ultrafast Two-Step Process in the Non-Adiabatic Relaxation of the CH_2NH_2 Molecule. *Mol. Phys.* **104,** 1053–1060. doi:10.1080/00268970500417945 (2006).
2. Tapavicza, E., Tavernelli, I. & Rothlisberger, U. Trajectory Surface Hopping within Linear Response Time-Dependent Density-Functional Theory. *Phys. Rev. Lett.* **98,** 023001. doi:10.1103/PhysRevLett.98.023001 (2 Jan. 2007).
3. Tavernelli, I., Tapavicza, E. & Rothlisberger, U. Nonadiabatic Coupling Vectors within Linear Response Time-Dependent Density Functional Theory. *J. Chem. Phys.* **130,** 124107. doi:10.1063/1.3097192 (2009).
4. Tavernelli, I., Tapavicza, E. & Rothlisberger, U. Non-Adiabatic Dynamics using Time-Dependent Density Functional Theory: Assessing the Coupling Strengths. *J. Mol. Struct.: THEOCHEM* **914,** 22–29. doi:https://doi.org/10.1016/j.theochem.2009.04.020 (2009).
5. Westermayr, J., Gastegger, M., Vörös, D., Panzenboeck, L., Joerg, F., González, L. & Marquetand, P. Deep learning study of tyrosine reveals that roaming can lead to photodamage. *Nat. Chem.* **14,** 914–919. doi:10.1038/s41557-022-00950-z (2022).
6. Mitri ć, R., Petersen, J. & Bona či ć-Koutecký, V. Laser-Field-Induced Surface-Hopping Method for the Simulation and Control of Ultrafast Photodynamics. *Phys. Rev. A* **79,** 053416. doi:10.1103/PhysRevA.79.053416 (5 May 2009).
7. Horton, S. L., Liu, Y., Forbes, R., Makhija, V., Lausten, R., Stolow, A., Hockett, P., Marquetand, P., Rozgonyi, T. & Weinacht, T. Excited state dynamics of CH_2I_2 and CH_2BrI studied with UV pump VUV probe photoelectron spectroscopy. *J. Chem. Phys.* **150,** 174201. doi:10.1063/1.5086665 (2019).
8. Mai, S., Atkins, A. J., Plasser, F. & González, L. The Influence of the Electronic Structure Method on Intersystem Crossing Dynamics. The Case of Thioformaldehyde. *J. Chem. Theory Comput.* **15,** 3470–3480. doi:10.1021/acs.jctc.9b00282 (2019).
